# The Lateral Control of Unmanned Vehicles Based on Neural Network Identification and a Fast Tube Model Predictive Control Algorithm

**DOI:** 10.3390/s26061973

**Published:** 2026-03-21

**Authors:** Yong Dai, Zhichen Zhou

**Affiliations:** School of Automation and Electrical Engineering, Shenyang Ligong University, Shenyang 110159, China

**Keywords:** control-affine feedforward neural network (CAFNN), tube model predictive control (tube-MPC), linear complementarity problem (LCP), sliding mode control (SMC)

## Abstract

In traditional vehicle trajectory tracking processes, the dynamic model of the vehicle may not accurately represent complex and nonlinear vehicle behaviors. Moreover, conventional control methods may perform poorly when dealing with system uncertainties and disturbances, facing challenges in real-time computation. To address these issues, this paper proposes an autonomous driving control method based on control-affine feedforward neural network (CAFNN) and fast tube model predictive control (tube-MPC). This method utilizes CAFNN for system dynamic identification, replacing traditional mathematical modeling with data-driven neural network pattern recognition to more accurately describe the vehicle’s nonlinear dynamic characteristics. On this basis, the proposed tube-MPC structure is divided into two parts: nominal MPC and sliding mode control (SMC). The nominal MPC controller associates the MPC problem with a linear complementarity problem (LCP) using a ramp function, enabling rapid computation of the quadratic programming (QP) solution through piecewise affine (PWA) functions; the auxiliary SMC controller employs multi-power sliding mode reaching laws to enhance the system’s robustness against external disturbances and model uncertainties. This control strategy demonstrates high accuracy and stability in vehicle trajectory tracking under complex road conditions, providing strong support for the advancement of autonomous driving technology.

## 1. Introduction

Autonomous driving technology, a core component of modern intelligent transportation systems, relies critically on achieving high-precision and reliable vehicle trajectory tracking control. To realize high-precision tracking, it is essential to establish accurate models that capture the strongly nonlinear characteristics of vehicle systems and to design efficient, robust control strategies [1]. Common trajectory tracking control methods [2] include proportional-integral-derivative (PID) control, model predictive control (MPC), sliding mode control (SMC), adaptive control, linear quadratic regulator (LQR), pure pursuit control, and neural network-based control.

To mitigate the impact of time-varying model parameters and system disturbances on control performance, Liang et al. proposed a continuous-time solution scheme for active front steering technology applied to autonomous path tracking. This scheme integrates robust MPC, a non-parallel distributed compensation method, and a polytopic model, with experiments verifying its effectiveness in enhancing path tracking performance [3]. Chu et al. developed a trajectory planning and tracking framework that uses artificial potential fields to generate target trajectories and employs PID feedback for tracking [4]. However, PID control performance is limited in dynamic environments, often requiring integration with more complex methods or parameter optimization to meet precise control demands. Shi et al. introduced an intelligent vehicle path tracking method based on improved MPC hybrid PID control theory, designing hybrid PID controllers for different road conditions and maintaining the yaw angle within $-4^{{}^{\circ}}$ to $2^{{}^{\circ}}$ at medium-to-high speeds, thus significantly improving tracking accuracy [5]. Sun et al., based on the Grünwald-Letnikov definition of fractional-order differences for tracking errors, designed a novel discrete fractional-order sliding mode surface, which effectively improved the tracking performance of linear control systems and ensured control objectives were met [6]. While SMC offers strong performance, chattering and discontinuity remain its primary drawbacks. To address lateral deviation and stability issues during tire slip, Hu et al. designed a novel output-constrained controller that combines an adaptive mechanism with linear quadratic control to achieve optimal active front steering angle and direct yaw moment control [7]. Its core relies on online learning and real-time adjustment, but this introduces a substantial real-time computational burden. Yang et al. used a 2-degree-of-freedom (DOF) vehicle dynamics model to construct a tracking error model, designing separate feedforward and predictive LQR controllers to reduce tracking error. Simulations and hardware-in-the-loop tests validated the algorithm’s practical effectiveness in winding intersections and complex steering conditions [8]. However, LQR control is limited by its reliance on linear systems and accurate state-space models, requiring extensions when applied to nonlinear systems or scenarios with input constraints. Ahn et al. selected preview points by analyzing the geometric relationship between the vehicle and the path, applying pure pursuit control to path tracking in complex and narrow road environments [9]. Pure pursuit performance degrades at high speeds or under complex road conditions, necessitating further optimization to improve robustness and accuracy. BMW designed a vehicle sideslip angle estimation model combining deep neural networks with sensor data fusion, which exhibits stronger adaptive characteristics, overcomes challenges such as time synchronization errors, and improves system accuracy and stability [10].

Vehicle trajectory tracking control often requires balancing rapid response with stable maintenance [11]. For example, Rodriguez et al. proposed a strategy combining MPC and LQR, which effectively reduces the computational complexity of the MPC optimization problem while enhancing system execution efficiency and smoothness [12]. Additionally, Su et al. introduced a novel neural network adaptive sliding mode controller, which significantly suppresses sliding mode chattering by integrating a radial basis function neural network, thereby enhancing the system’s compensation capability for uncertainties and external disturbances [13]. However, purely adaptive control methods, while aiming to handle changes through online learning, are often constrained by inherent drawbacks such as real-time computational burden, poor transient performance before parameter convergence, and reliance on persistent excitation conditions. Therefore, the current research trend leans more towards integrating adaptive concepts with other methods that possess more robust frameworks.

Against this backdrop, the MPC method is highly favored for its ability to explicitly handle multivariate, multi-constraint optimization problems [14,15], and it naturally possesses a certain degree of robustness. Furthermore, this method can impose constraints on system states, thereby ensuring driving safety and stability. To enhance the system’s adaptability to uncertainties, robust MPC methods such as tube-MPC have been developed. This method uses feedback control to keep the system state within a predefined "tube" maintaining performance even under model uncertainties and disturbances [16].

However, traditional modeling and control methods still have limitations when dealing with complex vehicle dynamics [17]. Therefore, research combining data-driven modeling with advanced control algorithms has gained significant attention in recent years. For example, Spielberg et al. employed neural networks to replace traditional physical models, achieving effective prediction even under harsh road conditions [18]. Additionally, Rokonuzzaman et al. researched the integration of neural networks with MPC, striking a better balance between control accuracy and computational burden. Their proposed offline neural network MPC demonstrated stronger adaptability compared to traditional MPC [19]. Recently, Liang et al. proposed an integrated deep reinforcement learning framework to enhance the high-speed cruising performance of autonomous vehicles in mixed traffic scenarios [20], this framework synthesizes behavioral decision-making, path planning, and motion control modules to achieve adaptive decision-making and human-like lane-changing path planning for autonomous vehicles. This work further verifies the excellent application potential of intelligent learning algorithms in improving the lateral control performance of autonomous vehicles under high-speed complex working conditions, and provides a valuable research direction for the fusion of learning-based methods and robust control strategies in the field of vehicle lateral control.

Although the aforementioned methods have made significant progress, challenges remain in terms of real-time computational efficiency and solution speed [21]. Therefore, the innovations of this paper are as follows:

(1) A vehicle kinematic identification method based on a control-affine feedforward neural network (CAFNN) is proposed, which can more accurately capture the nonlinear dynamic characteristics of vehicles. On this basis, a tube-MPC framework is introduced to enhance system robustness.
(2) Addressing the difficulty of real-time solving large-scale quadratic programming (QP) problems in MPC, this paper transforms the MPC problem into a linear complementarity problem (LCP) using the ramp function [22]. By reconstructing the optimization problem based on the Karush-Kuhn-Tucker (KKT) conditions and leveraging a piecewise affine (PWA) analytical form, computational efficiency is significantly improved.
(3) To further counteract external disturbances and model uncertainties, an auxiliary sliding mode controller based on a multi-power reaching law is designed, effectively enhancing the system’s robustness under nonlinear disturbances.

## 2. System Modeling and CAFNN Model

### 2.1. Vehicle Model

**Figure 1:**
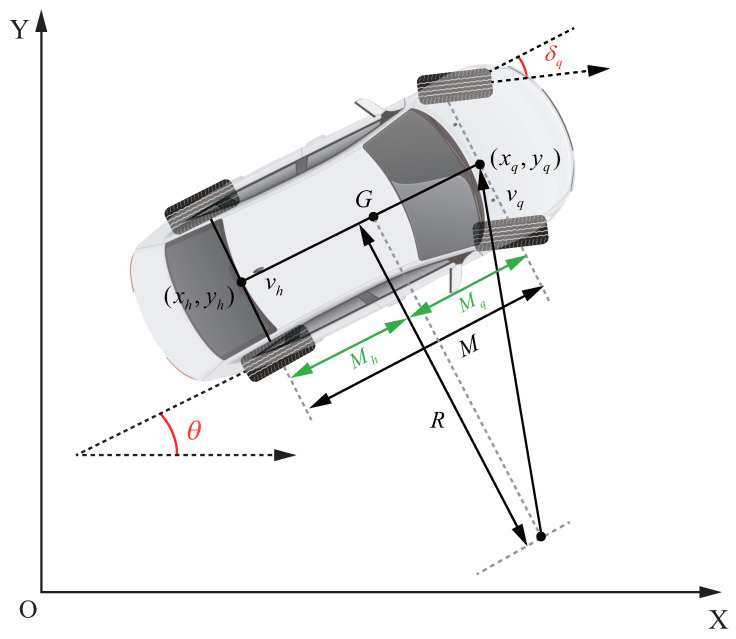
Vehicle kinematic model.

To simplify the vehicle model, the following assumption is made: the vehicle is a two-dimensional planar moving object. Therefore, a 2-DOF kinematic model is used to describe the kinematic characteristics of the vehicle. Figure 1 illustrates a typical 2-DOF vehicle steering kinematic model.

Where $\left(x_{h},y_{h}\right)$ and $\left(x_{q},y_{q}\right)$ represent the coordinates of the rear axle and front axle, respectively. *M* denotes the wheelbase length. $\delta_{q}$ represents the front wheel steering angle. θ denotes the vehicle heading angle. $v_{q}$ represents the front wheel speed. $v_{h}$ represents the rear wheel speed. *R* represents the turning radius of the rear wheel, which can be defined as the radius of the road curvature. The modeling and real-time control of the control system in this paper are both based on this kinematic model. The kinematic relationship between the front and rear axles can be expressed as:

(1) $$\left\{\begin{array}{c} x_{q}=x_{h}+M\cos\theta \\ y_{q}=y_{h}+M\sin\theta \end{array},\right.$$

at the rear axle travel pivot point ($x_{h}$, $y_{h}$), the velocity is

(2) $$v_{h}={\dot{x}}_{h}\cos\theta +{\dot{y}}_{h}\sin\theta .$$

Assuming that the vehicle’s sideslip angle remains constant during the steering process, the kinematic relationship between the front and rear axles is

(3) $$\left\{\begin{array}{c} {\dot{x}}_{h}\sin\theta -{\dot{y}}_{h}\cos\theta =0\\ {\dot{x}}_{q}\sin\left(\theta +\delta_{q}\right)-{\dot{y}}_{q}\cos\left(\theta +\delta_{q}\right)=0 \end{array}.\right.$$

Combining Equations (1) and (2), we obtain

(4) $$\left\{\begin{array}{c} {\dot{x}}_{h}=v_{h}\cos\theta \\ {\dot{y}}_{h}=v_{h}\sin\theta \end{array},\right.$$

where *w* is the vehicle yaw rate. *R* is the turning radius. $\delta_{q}$ is the front wheel steering angle, which can be defined as:

(5) $$\omega =\frac{v_{h}}{M}\tan\delta_{q}.$$

Meanwhile, from ω and the vehicle speed $v_{h}$, we can obtain

(6) $$\left\{\begin{array}{c} R=v_{h}/\omega \\ \delta_{q}=\arctan\left(M/R\right) \end{array}.\right.$$

By combining Equations (4) and (6), the vehicle kinematic model can be written as:

(7) $$\left[\begin{array}{c} {\dot{x}}_{h}\\ {\dot{y}}_{h}\\ \dot{\theta } \end{array}\right]=\left[\begin{array}{c} \cos\theta \\ \sin\theta \\ \tan\delta_{q}/M \end{array}\right]v_{h}.$$

During the process of trajectory tracking control, control inputs and state variables are used to define the vehicle’s kinematic model. Therefore, the model can be expressed as:

(8) $$\dot{\varepsilon }=f\left(\varepsilon ,u\right),$$

where the state variables are represented as $\varepsilon ={\left[x_{h},y_{h},\theta \right]}^{T}$, and the control inputs are represented as $u={\left[v_{h},\delta_{q}\right]}^{T}$.

The subscript *r* is used to define the various reference quantities of the vehicle kinematic model. The state variables and control inputs of the desired trajectory satisfy the following relationship at any given moment:

(9) $${\dot{\varepsilon }}_{r}=f\left(\varepsilon_{r},u_{r}\right),$$

where $\varepsilon_{r}={\left[x_{r},y_{r},\theta_{r}\right]}^{T}$, $u_{r}={\left[v_{r},\delta_{r}\right]}^{T}$. Expanding the equation using Taylor series at any point $\left(\varepsilon_{r},u_{r}\right)$ and neglecting higher-order terms yields:

(10) $${\dot{\varepsilon }}_{r}=f\left(\varepsilon_{r},u_{r}\right)+{\left.\frac{\partial f(\varepsilon ,u)}{\partial \varepsilon }\right|}_{\begin{array}{c} \varepsilon =\varepsilon_{r}\\ u=u_{r} \end{array}}\left(\varepsilon -\varepsilon_{r}\right)+{\left.\frac{\partial f(\varepsilon ,u)}{\partial u}\right|}_{\begin{array}{c} \varepsilon =\varepsilon_{r}\\ u=u_{r} \end{array}}\left(u-u_{r}\right).$$

Subtracting Equation (9) from Equation (10) yields the continuous-time linear model of the system:

(11) $$\dot{\hat{\varepsilon }}=\left[\begin{array}{c} {\dot{x}}_{h}-{\dot{x}}_{r}\\ {\dot{y}}_{h}-{\dot{y}}_{r}\\ \dot{\theta }-\dot{\theta }r \end{array}\right]=\left[\begin{array}{ccc} 0 & 0 & -v_{r}\sin\theta_{r}\\ 0 & 0 & v_{r}\cos\theta_{r}\\ 0 & 0 & 0 \end{array}\right]\left[\begin{array}{c} x_{h}-x_{r}\\ y_{h}-y_{r}\\ \theta -\theta_{r} \end{array}\right]+\left[\begin{array}{cc} \cos\theta_{r} & 0\\ \sin\theta_{r} & 0\\ \frac{\tan\delta_{r}}{M} & \frac{v_{r}}{M\cos^{2}\delta_{r}} \end{array}\right]\left[\begin{array}{c} v_{h}-v_{r}\\ \delta q-\delta_{r} \end{array}\right].$$

The linearized Equation (11) is discretized. In the discrete system, the current time step is denoted as *k*, and the next time step is denoted as k+1. The linear discrete-time model is as follows:

(12) $$\hat{\varepsilon }\left(k+1\right)=A_{k,t}\hat{\varepsilon }\left(k\right)+B_{k,t}\hat{u}\left(k\right),$$

where,

(13) $$\hat{\varepsilon }\left(k\right)=\varepsilon \left(k\right)-\varepsilon_{r}\left(k\right)=\left[\begin{array}{c} x_{h}\left(k\right)-x_{r}\left(k\right)\\ y_{h}\left(k\right)-y_{r}\left(k\right)\\ \theta \left(k\right)-\theta_{r}\left(k\right) \end{array}\right],$$

(14) $$\hat{u}\left(k\right)=u\left(k\right)-u_{r}\left(k\right)=\left[\begin{array}{c} v_{h}\left(k\right)-v_{r}\left(k\right)\\ \delta q\left(k\right)-\delta_{r}\left(k\right) \end{array}\right],$$

(15) $$A_{k,t}=I+T\cdot A=\left[\begin{array}{ccc} 1 & 0 & -V_{r}\sin\theta_{r}T\\ 0 & 1 & V_{r}\cos\theta_{r}T\\ 0 & 0 & 1 \end{array}\right],$$

(16) $$B_{k,t}=T\cdot B=\left[\begin{array}{cc} \cos\theta_{r}T & 0\\ \sin\theta_{r}T & 0\\ \frac{\tan\delta_{r}T}{M} & \frac{V_{r}T}{M\cos^{2}\delta_{r}} \end{array}\right],$$

where $A_{k,t}$ is the discretized system state error dynamic matrix, which incorporates the influence of the reference speed $V_{r}$ and the reference heading angle $\theta_{r}$. $B_{k,t}$ is the discretized system control matrix, describing how control inputs (such as speed $V_{r}$ and steering angle $\delta_{r}$) affect the system state error. Through these relationships, the control system can more effectively track the vehicle’s reference trajectory and make corresponding control adjustments.

The simplified assumptions (e.g., constant centroid sideslip angle) in the 2-DOF vehicle kinematic model will introduce certain modeling inaccuracies, which are fully captured and compensated by the subsequent CAFNN identification process. The CAFNN takes the state and control inputs of this simplified model as the input layer, and fits the deviation between the theoretical output of the simplified model and the actual motion state of the vehicle through training on real vehicle test data covering tire dynamics, high-speed nonlinear characteristics and other unmodeled dynamics, thus making up for the modeling inaccuracies caused by simplified assumptions. In addition, the auxiliary sliding mode controller in the tube-MPC framework further suppresses the comprehensive uncertainty composed of modeling inaccuracies and external disturbances, which ensures that the modeling simplification will not affect the final control performance of the system.

### 2.2. CAFNN Model

For complex vehicle trajectory tracking control problems, the CAFNN is employed to model the system based on the control affine form, which is consistent with the core dynamic characteristics of the actual vehicle. This modeling method ensures high-precision tracking of the reference trajectory by fitting the nonlinear dynamic characteristics of the vehicle while maintaining the structural advantage of the affine system for easy controller design. Consider the following affine system control process [23] that matches the vehicle dynamic characteristics:

(17) x(k+1)=f(x(k))x(k)+g(x(k))u(k),

where k=0,1,2… represents the control cycle, $x\left(k\right)={\left[x_{h}\left(k\right),y_{h}\left(k\right),\theta \left(k\right)\right]}^{T}\in X$ is the $n_{x}\times 1$ dimensional state vector, and $u_{k}={\left[v_{h}\left(k\right),\delta_{q}\left(k\right)\right]}^{T}\in U$ is the $n_{u}\times 1$ dimensional control vector. Moreover, the state vector here is actually a unified discretized representation of the state vector of the vehicle kinematic model presented earlier.

(18) $$f\left(x\left(k\right)\right)=\left[\begin{array}{ccc} f_{11}\left(x\left(k\right)\right) & \cdots & f_{1n_{x}}\left(x\left(k\right)\right)\\ \vdots & \ddots & \vdots \\ f_{n_{x}1}\left(x\left(k\right)\right) & \cdots & f_{n_{x}n_{x}}\left(x\left(k\right)\right) \end{array}\right],$$

(19) $$g\left(x\left(k\right)\right)=\left[\begin{array}{ccc} g_{11}\left(x\left(k\right)\right) & \cdots & g_{1n_{u}}\left(x\left(k\right)\right)\\ \vdots & \ddots & \vdots \\ g_{n_{x}1}\left(x\left(k\right)\right) & \cdots & g_{n_{x}n_{u}}\left(x\left(k\right)\right) \end{array}\right].$$

**Figure 2:**
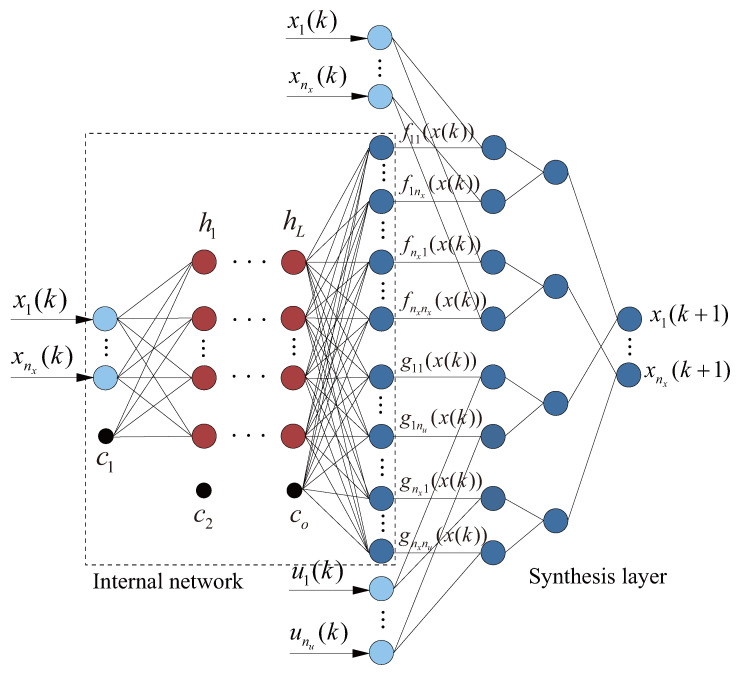
CAFNN network architecture.

From Figure 2, the CAFNN comprises an internal network and a synthesis layer: the former identifies f(·) and g(·), while the latter computes the network output via its tailored structural design. The internal network takes the current state vector x(k) at time step *k* as input, and outputs the corresponding matrices f(x(k)) and g(x(k)).

Within the internal network, there are *L* hidden layers, and the output of each layer can be derived as:

(20) $$h_{i}=\tanh\left(W_{i}h_{i-1}+c_{i}\right),$$

where $h_{i}\left(i\in \left[1,L\right]\right)$ represents the output of the *i*-th hidden layer, tanh is a commonly used activation function in neural networks, $W_{i}$ represents the connection weights between the current hidden layer and the previous hidden layer, and $c_{i}$ denotes the connection thresholds between neurons. The output of the internal network, together with the control vector u(k), forms the input to the synthesis layer. Finally, the synthesis layer computes the state vector x(k+1) at the (k+1)-th time step.

**Remark 1.**  *The kinematic model established in this paper is simplified to adapt to the computing power requirements of real-time control, focusing on the core geometric constraints of the vehicle’s planar motion and excluding tire dynamics and high-speed nonlinear dynamics. These characteristics and modeling errors are specifically captured and characterized by the CAFNN. Based on the state and control inputs of the kinematic model, the CAFNN is trained on datasets covering tire and high-speed nonlinear characteristics under different vehicle speeds and road conditions. It captures the relevant nonlinear laws by fitting the deviation between the theoretical output of the model and the actual motion state of the real vehicle, and collaborates with the kinematic model to supplement the characterization of the uncovered nonlinear dynamic characteristics. This addresses the problem of incomplete modeling and provides an accurate dynamic foundation for the fast tube-MPC.*

In this study, the root mean square error (RMSE) is employed as the loss function to measure the deviation between the network’s predicted output and the actual kinematic data. The RMSE loss function is defined as follows:

(21) $$\mathrm{RMSE}=\sqrt{\frac{1}{n}\sum_{i=1}^{n}{\left(x_{NN}\left(i\right)-x\left(i\right)\right)}^{2}}.$$

To balance model complexity and performance, the optimal network architecture of the CAFNN is determined by minimizing its performance metric $J_{NN}$. The designed objective function is as follows:

(22) $$\min J_{NN}=\frac{1}{2}\left(\tau_{1}\mathrm{RMSE}{\left(L\right)}^{2}+\tau_{2}L^{2}\right),$$

where the two parameters $\tau_{1}$ and $\tau_{2}$ are the weight coefficients of the RMSE and the number of hidden layers *L* in the loss function, respectively. By adjusting these two parameters, the complexity and prediction accuracy of the model can be balanced. In this study, multiple experiments were conducted on the CAFNN, and ultimately a network structure with 3 hidden layers was selected. The specific selection was determined by minimizing the RMSE loss function. Each hidden layer contains 64 neurons, with tanh as the activation function. This architecture helps capture the nonlinear kinematic characteristics of the vehicle.

To quantify the identification accuracy of the offline-trained CAFNN model for vehicle nonlinear motion characteristics, the key quantitative indicators from the model training and testing phases are presented here. The data originates from 1000 sets of kinematic data collected during real vehicle experiments, with the specific results shown in Table 1. The comprehensive $R^{2}$ of the CAFNN model on the test set reaches 0.972, indicating that it can explain 97.2% of the changes in vehicle motion states. This validates its high-precision identification capability for vehicle nonlinear motion characteristics.

To demonstrate the applicability and superiority of the CAFNN model, this paper selects two types of simple benchmark models commonly used in the field of autonomous driving for comparison. The results are shown in Table 2. The CAFNN model exhibits the best performance in terms of identification accuracy, computational efficiency, and adaptability.

To ensure the controllability of the learned g(x) and the invertibility of $Cg_{NN}$, a dual constraint strategy was applied during CAFNN training: hard constraints were imposed on the output $g_{NN}\left(x\right)$ matrix to keep the determinant of $Cg_{NN}$ non-zero, satisfying the mathematical invertibility condition; a controllability criterion regularizer was added to the loss function to constrain the rank of g(x) for controllability, and the full-condition real-vehicle excitation data guaranteed the learned g(x) covers the actual controllable state space of the vehicle.

The modeling approach for the vehicle model has been thoroughly discussed. Building upon this foundation, the following sections will focus on the design of the control algorithm, providing a detailed elaboration of the novel control framework proposed in this paper.

## 3. Tube-MPC Controller Design

Based on the CAFNN model and considering the vehicle operating mode, the neural network model can be reformulated as the nominal model of the system under ideal conditions as follows:

(23) $$x_{NN}\left(k+1\right)=f_{NN}\left(x_{NN}\left(k\right)\right)x_{NN}\left(k\right)+g_{NN}\left(x_{NN}\left(k\right)\right)u_{NN}\left(k\right).$$

The modeling error of the CAFNN is defined as the unknown disturbance experienced by the vehicle during operation. Combing with the external unknown disturbances, the comprehensive uncertainty disturbance set W is defined as a bounded closed set in the $n_{x}$-dimensional real space $R^{n_{x}}$. The disturbance term ω(k) caused by the comprehensive uncertainty satisfies ω(k)∈W, and the disturbance set W is rigorously defined as: $W=\left\{\omega \left(k\right)\in R^{n_{x}}|∥\omega \left(k\right)∥\leq \bar{\omega },\;\forall k\in N\right\}$. Define the reference trajectory as $x_{r}\left(k\right)$. Then, the nominal tracking error can be expressed as:

(24) $$e_{NN}\left(k\right)=x_{NN}\left(k\right)-x_{r}\left(k\right).$$

Based on the above definition, the actual system considering comprehensive uncertainty is then defined as:

(25) $$x\left(k+1\right)=f_{NN}\left(x\left(k\right)\right)x\left(k\right)+g_{NN}\left(x\left(k\right)\right)u\left(k\right)+\omega \left(k\right).$$

Now, let’s define error system, let the total control law be

(26) $$u\left(k\right)=u_{NN}\left(k\right)+\kappa \eta \left(k\right),$$

then,

(27) $$e\left(k+1\right)=f_{NN}e\left(k\right)+g_{NN}\kappa \eta \left(k\right)+\omega \left(k\right),$$

where $e\left(k\right)=x\left(k\right)-e_{NN}\left(k\right)$ represents the error state between the actual system and the nominal system. u(k) is the total control input of the actual system at time step *k*, $u_{NN}\left(k\right)$ is the nominal control input at time step *k*, which is the control law of the nominal MPC under the CAFNN model, $u_{NN}\left(k\right)\in U$ represents the constraint on the nominal system input, κ is a two-dimensional column vector used to adjust the gain of the influence of the error term η(k) on the total control input, and η(k) is the auxiliary SMC control law, representing the deviation between the nominal model and the actual system dynamics.

Next, we will discuss the control algorithm in two parts: first, we will elaborate on the design of the nominal MPC controller, its combination of LCP and QP-MPC, as well as the corresponding stability analysis; then, we will address the design of the auxiliary SMC controller and its stability analysis.

### 3.1. Nominal Controller

#### 3.1.1. MPC Design

In the absence of disturbance effects on the system, the nominal controller is designed as follows:

(28) $$e_{NN}\left(k+1\right)=f_{NN}e_{NN}\left(k\right)+g_{NN}u_{NN}\left(k\right),$$

based on the state equation of the nominal system at the current time *k*, the system states from future time k+1 to $k+N_{p}$ can be predicted, yielding:

(29) $$\left\{\begin{array}{c} e_{NN}\left(k+1\right)=f_{NN}e_{NN}\left(k\right)+g_{NN}u_{NN}\left(k\right)\\ e_{NN}\left(k+2\right)={f_{NN}}^{2}e_{NN}\left(k\right)+f_{NN}g_{NN}u_{NN}\left(k\right)+g_{NN}u_{NN}\left(k+1\right)\\ \;\vdots \\ e_{NN}\left(k+N_{c}\right)={f_{NN}}^{N_{c}}e_{NN}\left(k\right)+{f_{NN}}^{N_{c}-1}g_{NN}u_{NN}\left(k\right)+\cdots +g_{NN}u_{NN}\left(k+N_{c}-1\right)\\ \;\vdots \\ e_{NN}\left(k+N_{p}\right)={f_{NN}}^{N_{p}}e_{NN}\left(k\right)+{f_{NN}}^{N_{p}-1}g_{NN}u_{NN}\left(k\right)+\cdots +{f_{NN}}^{N_{p}-N_{c}}g_{NN}u_{NN}\left(k+N_{c}-1\right) \end{array}.\right.$$

The core of the fast tube-MPC proposed in this paper is to transform the QP problem into an LCP using the ramp function and solve it quickly through the PWA analytical form. The actual engineering solution process is divided into five key steps, briefly outlined as follows: (a) Optimization problem modeling; (b) Variable substitution and problem simplification; (c) Transformation into LCP; (d) PWA analytical solution; (e) Control output and rolling optimization. The detailed derivation process is presented next.

At each sampling interval, MPC optimization is performed based on the CAFNN model. During the system tracking process, to ensure that the control output converges toward its reference value at the end of the prediction horizon, thereby achieving accurate tracking of the target path, an optimal control approach is implemented by defining a quadratic cost function:

(30) $$J\left(k\right)=\sum_{i=1}^{N_{p}-1}{∥e_{NN}\left(k+i|k\right)∥}_{Q}^{2}+\sum_{i=0}^{N_{c}-1}{∥u_{NN}\left(k+i|k\right)∥}_{R}^{2}+{∥e_{NN}\left(k+N_{p}|k\right)∥}_{P}^{2},$$

where ${∥e_{NN}\left(k+i|k\right)∥}_{Q}^{2}$ reflects the nominal system’s ability to follow the reference trajectory. ${∥u_{NN}\left(k+i|k\right)∥}_{R}^{2}$ reflects the constraints on changes in control inputs. $N_{p}$ is the prediction horizon, and $N_{c}$ is the control horizon. *Q* and *R* are weight matrices used to adjust the influence of state variables and control inputs. ${∥e_{NN}\left(k+i|k\right)∥}_{P}^{2}$ is referred to as the terminal penalty term, and *P* is called the terminal penalty matrix. The above objective function can be reformulated as follows:

(31) $$\min_{U_{NN}\left(k\right)}J\left(k\right)=\min\left\{{∥E_{NN}∥}_{Q}^{2}+{∥U_{NN}∥}_{R}^{2}+{∥E_{NN}∥}_{P}^{2}\right\},$$

(32) $$E_{NN}\left(k\right)=F_{NN}\left(k\right)e_{NN}\left(k\right)+G_{NN}\left(k\right)u_{NN}\left(k\right),$$

where,

(33) $$E_{NN}={\left[e_{NN}{\left(k+1\right)}^{T},e_{NN}{\left(k+2\right)}^{T},\cdots ,e_{NN}{\left(k+N_{c}\right)}^{T},\cdots ,e_{NN}{\left(k+N_{p}\right)}^{T}\right]}^{T},$$

(34) $$F_{NN}={\left[f_{NN}^{T},{f_{NN}^{2}}^{T},\cdots ,{f_{NN}^{Nc}}^{T},\cdots ,{f_{NN}^{Np}}^{T}\right]}^{T},$$

(35) $$G_{NN}=\left[\begin{array}{ccccc} g_{NN} & 0 & 0 & \cdots & 0\\ f_{NN}g_{NN} & g_{NN} & 0 & \cdots & 0\\ \vdots & \vdots & \ddots & \ddots & \vdots \\ f_{NN}^{N_{c}-1}g_{NN} & f_{NN}^{Nc-2}g_{NN} & \cdots & \cdots & g_{NN}\\ \vdots & \vdots & \ddots & \ddots & \vdots \\ f_{NN}^{Np-1}g_{NN} & f_{NN}^{Np-1}g_{NN} & \cdots & \cdots & f_{NN}^{Np-N_{C}}g_{NN} \end{array}\right],$$

(36) $$U_{NN}={\left[u_{NN}{\left(k\right)}^{T},u_{NN}{\left(k+1\right)}^{T},\cdots ,u_{NN}{\left(k+N_{c}-1\right)}^{T}\right]}^{T},$$

where the column vector $U_{NN}={\left[u_{NN}^{T}\left(k\right),\cdots ,u_{NN}^{T}\left(k+N_{c}-1\right)\right]}^{T}$, and since $Q\in S^{n}\geq 0$, $R\in S^{n}>0$, $\forall i=1,\ldots ,N_{p}-1$, it follows that $P\in S^{n}\geq 0$. The MPC problem can be expressed in the following quadratic programming form:

(37) $$\begin{array}{cc} & \underset{U_{NN}}{\mathrm{minimize}}\frac{1}{2}{U_{NN}}^{T}HU_{NN}+e_{NN}\left(k\right)FU_{NN}^{T}, \end{array}$$

where *H* and *F* are matrix functions of $f_{NN}$, $g_{NN}$, *Q* and *R*, respectively, see the paper [24] for details.

#### 3.1.2. Combination of LCP and QP-MPC

The ramp function is defined as:

(38) $$r\left(y\right)=\left\{\begin{array}{cc} y, & \;\mathrm{if}\;y\geq 0\\ 0, & \;\mathrm{if}\;y<0 \end{array}\right..$$

**Lemma 1.**  *The unit ramp function satisfies the following functional relationship, ∀y∈R:*

(39) $$\left\{\begin{array}{c} (r(y)-y)r(y)=0\\ (r(y)-y)\geq 0\\ r(y)\geq 0 \end{array}.\right.$$

**Proof.**  First, express the ramp function as a convex optimization problem with parameter *y*, and the method for solving it is as follows:

(40) $$\left\{\begin{array}{c} \underset{r}{\mathrm{minimize}}\frac{1}{2}{\left(r-y\right)}^{2}\\ \;\mathrm{subject}\;\mathrm{to}\;r\geq 0 \end{array}.\right.$$

By leveraging the Lagrangian associated with the optimization problem, the KKT conditions are derived. Since the problem is strictly convex, these conditions are both necessary and sufficient for optimality. To characterize the variables (y,r), the following expressions can be utilized:

(41) $$L\left(r,\alpha \right)=\frac{1}{2}{\left(r-y\right)}^{2}-\alpha r.$$

Combining the complementary slackness condition and feasibility conditions:

(42) $$\left\{\begin{array}{c} (r-y)-\alpha =0\\ \alpha r=0\\ \alpha \geq 0\\ r\geq 0 \end{array},\right.$$

where $r\left(y\right)={\left[r\left(y_{1}\right),r\left(y_{2}\right),\ldots ,r\left(y_{n}\right)\right]}^{T}$ serves as a vector-valued ramp function defined over y∈R, the function r:R→R is the underlying definition.

Based on the approach in [22], the optimization problem in Equation (37) is solved using a ramp function, yielding the final result. The solution of the implicit equation which corresponds to the optimal solution of the QP problem can be derived as follows:

(43) $$U_{NN}=-H^{-1}F^{T}e_{NN}-H^{-1}G^{T}r\left(y\right).$$

where *G* is the parameter matrix and r(y) is the ramp function. By defining the ramp function, the solution process of the LCP can be simplified. □

To establish a strict Lyapunov non-increasing relationship, a standard MPC stability framework is adopted here: the terminal penalty matrix *P* is designed as the solution of the discrete algebraic Riccati equation for the nominal system, and the terminal constraint set is defined as the robust positive invariant set of the nominal system, ensuring recursive feasibility. The suboptimality ratio of the LCP-QP solution is bounded by a constant ρ∈(0,1), thus leading to $J_{L}\left(k+1\right)\leq \rho J_{L}\left(k\right)\leq J_{L}\left(k\right)$, which strictly proves the Lyapunov function decay and the asymptotic stability of the nominal system.

Here, we conduct quantitative comparative experiments on the computational efficiency of the LCP solution method proposed in this paper and the standard QP solution method. It can be clearly seen that the proposed method requires very short computation time, which fully verifies the superiority of the ramp function-based LCP method in terms of computational efficiency, as shown in Table 3.

#### 3.1.3. Stability Analysis of the Suboptimal MPC

This proof for the stability of suboptimal MPC is based on two core theoretical pillars consistent with the classic constrained MPC framework: (1) the suboptimal control law obtained by LCP-QP transformation is a feasible solution that strictly satisfies all state and input constraints of the vehicle system; (2) the Lyapunov function constructed by the MPC cost function has the monotonic non-increasing property for the suboptimal feasible solution. Combined with the compactness of the vehicle lateral control state space, the uniform ultimate boundedness and asymptotic stability of the closed-loop system are directly derived from the discrete Lyapunov stability theorem. The processing of summation and inequality in the following proof is all based on the above core logic, which ensures the rigor of the theoretical deduction for the conclusion that suboptimality can still guarantee closed-loop stability.

**Theorem 1.**  *Constrained MPC systems are fundamentally nonlinear, and stability is typically proven using Lyapunov’s theorem. The Lyapunov function is designed based on the cost function. Let the optimal cost function at time k be $J^{*}\left(k\right)$, expressed by the following equation:*

(44) $$V^{*}\left(k\right)=\sum_{i=1}^{N}{∥e_{NN}^{*}\left(k+i|k\right)∥}_{Q}^{2}+\sum_{i=0}^{N-1}{∥u_{NN}^{*}\left(k+i|k\right)∥}_{R}^{2}+{∥e_{NN}^{*}\left(k+N_{p}|k\right)∥}_{P}^{2},$$

*where $e_{NN}$ represents the optimal state variable under nominal system control, and $u_{NN}$ denotes the optimal control law of MPC. After combining QP and LCP, the control law obtained at a certain moment may be a suboptimal control law. The resulting stable control input is permissible, thereby ensuring the convergence and stability of the nominal system.*

**Proof.**  Based on the quadratic cost function under optimal control, the Lyapunov function can be designed as:

(45) $$u_{NN}^{*}\left(k\right)=u_{LNN}\left(k\right)+\Delta u_{NN}\left(k\right),$$

where $u_{LNN}\left(k\right)$ serves as the suboptimal control law obtained after combining QP and LCP, and $\Delta u_{NN}\left(k\right)$ represents the deviation between the optimal control law and the suboptimal control law. Under optimal control, the representation of the nominal system Equation (28) is as follows:

(46) $$e_{NN}^{*}\left(k+1\right)=f_{NN}e_{NN}^{*}\left(k\right)+g_{NN}u_{LNN}\left(k\right)+g_{NN}\Delta u_{NN}\left(k\right).$$

The nominal system under suboptimal control can be expressed as follows:

(47) $$e_{LNN}\left(k+1\right)=f_{NN}e_{LNN}\left(k\right)+g_{NN}u_{LNN}\left(k\right).$$

The deviation of the nominal system state variables under optimal control versus suboptimal control is expressed as:

(48) $$\Delta e_{NN}\left(k+1\right)=f_{NN}\Delta e_{NN}\left(k\right)+g_{NN}\Delta u_{NN}\left(k\right).$$

The Lyapunov function under suboptimal control is designed as:

(49) $$J_{L}\left(k\right)=\sum_{i=1}^{N}{∥e_{LNN}\left(k+i|k\right)∥}_{Q}^{2}+\sum_{i=0}^{N}{∥u_{LNN}\left(k+i|k\right)∥}_{R}^{2}+{∥e_{LNN}\left(k+N_{p}|k\right)∥}_{P}^{2}.$$

From the above equations, the suboptimal objective function at time k+1 can be obtained, and its relationship is derived as follows:

(50) $$\begin{array}{cc} J^{*}\left(k+1\right)\leq & J_{L}\left(k+1\right)+\sum_{i=1}^{N-1}{∥\Delta e_{NN}\left(k+i+1|k+1\right)∥}_{Q}^{2}+\sum_{i=0}^{N-1}{∥\Delta u_{NN}\left(k+i+1|k+1\right)∥}_{R}^{2}\\ = & J_{L}\left(k+1\right)+\sum_{i=2}^{N-1}{∥\Delta e_{NN}\left(k+i|k\right)∥}_{Q}^{2}+\sum_{i=1}^{N-1}{∥\Delta u_{NN}\left(k+i|k\right)∥}_{R}^{2}\\ = & \sum_{i=1}^{N-1}{∥e_{LNN}\left(k+i+1|k+1\right)∥}_{Q}^{2}+\sum_{i=0}^{N-1}{∥u_{LNN}\left(k+i+1|k+1\right)∥}_{R}^{2}+{∥e_{LNN}\left(k+N_{p}+1|k+1\right)∥}_{P}^{2}\\ & +\sum_{i=2}^{N-1}{∥\Delta e_{NN}\left(k+i|k\right)∥}_{Q}^{2}+\sum_{i=1}^{N-1}{∥\Delta u_{NN}\left(k+i|k\right)∥}_{R}^{2}\\ = & \sum_{i=2}^{N-1}{∥e_{LNN}\left(k+i|k\right)∥}_{Q}^{2}+\sum_{i=1}^{N-1}{∥u_{LNN}\left(k+i|k\right)∥}_{R}^{2}+{∥e_{LNN}\left(k+N_{p}|k\right)∥}_{P}^{2}\\ & +\sum_{i=2}^{N-1}{∥\Delta e_{NN}\left(k+i|k\right)∥}_{Q}^{2}+\sum_{i=1}^{N-1}{∥\Delta u_{NN}\left(k+i|k\right)∥}_{R}^{2}\\ = & \sum_{i=1}^{N-1}{∥e_{LNN}\left(k+i|k\right)∥}_{Q}^{2}+\sum_{i=0}^{N-1}{∥u_{LNN}\left(k+i|k\right)∥}_{R}^{2}+{∥e_{LNN}\left(k+N_{p}|k\right)∥}_{P}^{2}\\ & -{∥e_{LNN}\left(k+i|k\right)∥}_{Q}^{2}-{∥u_{LNN}\left(k+i|k\right)∥}_{R}^{2}+\sum_{i=1}^{N-1}{∥\Delta e_{NN}\left(k+i|k\right)∥}_{Q}^{2}+\sum_{i=0}^{N-1}{∥\Delta u_{NN}\left(k+i|k\right)∥}_{R}^{2}\\ & -{∥\Delta e_{NN}\left(k+i|k\right)∥}_{Q}^{2}-{∥\Delta u_{NN}\left(k+i|k\right)∥}_{R}^{2}\\ = & J_{L}\left(k\right)+\sum_{i=1}^{N-1}{∥\Delta e_{NN}\left(k+i|k\right)∥}_{Q}^{2}+\sum_{i=0}^{N-1}{∥\Delta u_{NN}\left(k+i|k\right)∥}_{R}^{2}-{∥e_{NN}^{*}\left(k+i|k\right)∥}_{Q}^{2}-{∥u_{NN}^{*}\left(k+i|k\right)∥}_{R}^{2} \end{array}.$$

Since ${∥e_{NN}^{*}\left(k+i|k\right)|}_{Q}^{2}+{∥u_{NN}^{*}\left(k+i|k\right)∥}_{R}^{2}\geq 0$, we obtain $J_{L}\left(k+1\right)\leq J_{L}\left(k\right)$, which completes the proof of system stability. It can be observed that even if the solution is not globally optimal, the suboptimal control input can still keep the nominal system bounded and achieve convergence and stability. □

### 3.2. Auxiliary SMC Controller

#### 3.2.1. Controller Design

To enable the states of the actual system to approximate those of the nominal CAFNN system, thereby achieving effective suppression of their deviation, a tube robust framework is employed to characterize the error system (27). Using state variables, the discrete-time switching function is constructed as follows:

(51) s(k)=Ce(k),

where *C* is the sliding surface coefficient and $Cg_{NN}$ is an invertible matrix.

Due to the uncertainty of ω(k), the disturbance estimation method is used to estimate the disturbance term. It is represented by the following equation:

(52) $$\omega \left(k-1\right)=e\left(k\right)-f_{NN}e\left(k-1\right)-g_{NN}\kappa \eta \left(k\right).$$

To reduce chattering and shorten the time to reach the sliding mode surface, a multi-power reaching law method is adopted. This approach ensures better control precision while improving convergence speed. The multi-power discrete-time sliding mode reaching law is designed as follows [25]:

(53) $$\begin{array}{cc} & s\left(k+1\right)=\left(1-qT\right)s\left(k\right)-\sum_{i=1}^{3}k_{i}{\left|s\left(k\right)\right|}^{\xi_{i}}\cdot \mathrm{sig}\left(s\left(k\right)\right)+\delta \left(k\right)\\ & \mathrm{sig}\left(s\left(k\right)\right)=\frac{s(k)}{|s(k)|+\vartheta },\;\xi_{1}=\alpha ,\xi_{2}=\beta ,\xi_{3}=\gamma \end{array}$$

where 0<1−qT<1, $k_{1},k_{2},k_{3}>0$, α>1, 0<β<1, ϑ is a very small number, and δ(k) represents the disturbance rate of change.

Combining the error system model with the switching function, and based on the designed reaching law and the estimated disturbance term, the expression for the auxiliary control law is computed as follows:

(54) $$\eta \left(k\right)={\left(Cg_{NN}\kappa \right)}^{-1}\left[\left(1-qT\right)s\left(k\right)-k_{1}{\left|s\left(k\right)\right|}^{\alpha }\frac{s(k)}{|s(k)|+\vartheta }-k_{2}{\left|s\left(k\right)\right|}^{\beta }\frac{s(k)}{|s(k)|+\vartheta }-k_{3}{\left|s\left(k\right)\right|}^{\gamma }\frac{s(k)}{|s(k)|+\vartheta }+\delta \left(k\right)-Cf_{NN}e\left(k\right)\right].$$

#### 3.2.2. Stability Analysis of the Proposed SMC

**Theorem 2.**  *The approach described in Equation (54) can be employed to confine e(k) within the minimal feasible region.*

**Proof.**  Please refer to [25] for a detailed proof, which will not be elaborated upon in this paper. □

**Remark 2.**  *In the tube-MPC framework, this paper defines the robust invariant set as the set of error constraints of the actual system state relative to the CAFNN nominal model state, denoted as $\chi_{\epsilon }=\left\{e\left(k\right)|∥e(k)∥\leq \epsilon ,e\left(k\right)=x\left(k\right)-x_{NN}\left(k\right)\right\}$, where ϵ is the error threshold determined based on the system modeling error, the upper bound of external disturbances, and the control precision of SMC. This set serves as the core constraint boundary of the tube structure and forms the robust tube that enables the actual system state to track the nominal system state. The construction of the robust invariant set is based on the premise of bounded system uncertainty and disturbances (i.e., modeling error ω(k)∈W, disturbance variation rate $\left|\delta (k)\right|\leq \delta^{*}$). By incorporating the convergence characteristics and stability analysis of the multi-power sliding mode reaching law, the bounded convergence region O of the sliding mode surface s(k) is solved. Through back-stepping, the maximum allowable fluctuation range of the error state e(k) is derived, thereby determining the boundary ϵ of the invariant set $\chi_{\epsilon }$.*

The auxiliary SMC controller proposed in this paper is embedded within the tube-MPC framework. Its core function is to compensate for CAFNN modeling errors and external disturbances under complex road conditions, ensuring that the system states are constrained within the preset “tube”. In terms of chattering suppression, a smooth function is adopted to replace the traditional sign function, balancing convergence speed and smoothness, thereby effectively improving control smoothness and robustness.

### 3.3. The Workflow of the Proposed Algorithm

**Figure 3:**
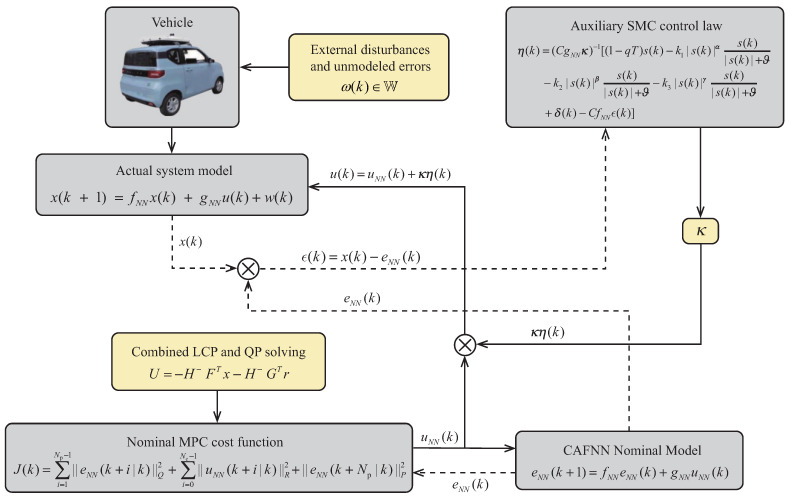
Algorithm control framework.

Figure 3 illustrates the control framework of the algorithm proposed in this paper. First, the nominal system state variable $e_{NN}\left(k\right)$ is obtained by identifying the vehicle kinematic model through a neural network. The actual system state variable x(k) is then derived by considering unmodeled uncertainties and unknown disturbances in the system.

Second, the nominal system state signal $e_{NN}\left(k\right)$ and the error system state signal e(k) together constitute the actual system state signal x(k). Next, based on the CAFNN nominal model, the complexity of solving the quadratic cost function optimization is reduced at the current moment by combining LCP with QP, thereby obtaining the nominal control law $u_{NN}\left(k\right)$.

Additionally, during the process of solving the optimization problem for the auxiliary SMC controller, the sliding surface and control law are designed to ensure that the actual system state approximates the nominal system state, confining the error fluctuations within a robust set.

Finally, the computed nominal control input $u_{NN}\left(k\right)$ and auxiliary control law η(k) are fed back to the vehicle as the ultimate actual control inputs, enabling the actual system trajectory to track the reference trajectory.

## 4. Simulations

To verify the real-time performance and robustness of the CAFNN-based dynamic model identification method and the improved fast tube-MPC control algorithm proposed in this paper in a simulation environment, this section designs a series of simulation experiments and validates them using a CarSim/Simulink co-simulation platform and a real vehicle test platform. In the simulation experiments, a three-dimensional vehicle simulation environment is built using CarSim and integrated with Simulink to execute the control algorithm and enable data interaction. Finally, the algorithm is practically validated on the real vehicle test platform, thereby comprehensively evaluating the dynamic performance and stability of the proposed method in complex driving scenarios. The indicators used in the experiment, such as yaw rate and sideslip angle, are vehicle dynamic performance evaluation metrics. They are solely used to verify the actual control effect of the control algorithm and are not related to the internal model design of the controller.

The specific experiments are conducted under the double lane-change condition and involve comparisons among multiple control algorithms, aiming to evaluate the comprehensive performance of the control algorithms. The double lane-change test condition is a classic method widely used for evaluating vehicle dynamic characteristics. Due to its excellent operability and representativeness, it is often regarded as an ideal test choice for verifying and optimizing the trajectory tracking control performance of intelligent driving vehicles. Based on this condition, the designed reference trajectory equation is as follows:

(55) $$Y_{\mathrm{ref}\;}\left(X\right)=\frac{d_{y1}}{2}\left(1+\tanh\left(z_{1}\right)\right)-\frac{d_{y2}}{2}\left(1+\tanh\left(z_{2}\right)\right),$$

(56) $$\varphi_{ref}\left(X\right)=\arctan\left(d_{y1}{\left(\frac{1}{\cosh\left(z_{1}\right)}\right)}^{2}\left(\frac{1.2}{d_{x1}}\right)-d_{y2}{\left(\frac{1}{\cosh\left(z_{2}\right)}\right)}^{2}\left(\frac{1.2}{d_{x2}}\right)\right),$$

the selected values are:

(57) $$\left\{\begin{array}{c} z_{1}=\frac{2.4}{25}\left(X-27.19\right)-1.2\\ z_{2}=\frac{2.4}{21.95}\left(X-56.46\right)-1.2\\ d_{x1}=25\\ d_{x2}=21.95\\ d_{y1}=4.05\\ d_{y2}=5.7 \end{array}.\right.$$

All computation time tests were conducted on the same hardware platform: Intel Core i7-12700H, 32 GB RAM, Windows 10. The algorithm was implemented in Simulink; the traditional MPC was solved by Gurobi 9.5, and the proposed tube-MPC was implemented with a custom LCP solver. To ensure consistent timing conditions, no thermal start was adopted for all methods.

The nominal and auxiliary controller parameters are set as shown in Table 4. Each parameter is the optimal one derived from multiple tests, and these parameters ensure the reproducibility of the experiments.

**Figure 4:**
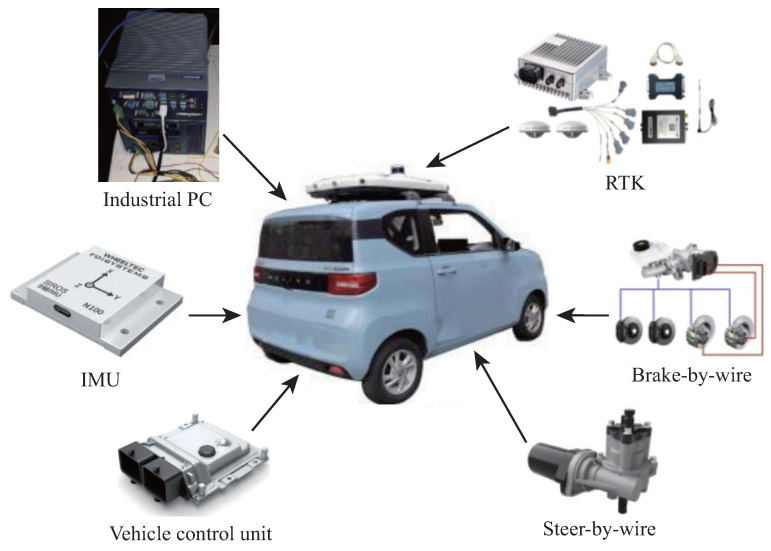
Real vehicle driving platform.

The physical vehicle test platform is shown in Figure 4. This platform features an automotive-grade chassis and full-stack by-wire control capabilities, enabling key functions such as front-wheel Ackermann steering, rear-wheel electric drive, and electro-hydraulic braking, providing foundational control support for autonomous driving systems. The platform also integrates modules such as an inertial measurement unit (IMU), a real-time kinematic (RTK) positioning system, and a by-wire steering system. Efficient data acquisition and signal processing are achieved via the CAN-BUS, ensuring real-time control accuracy and stability for autonomous vehicles. The basic parameter information of the vehicle is presented in Table 5.

### 4.1. Simulation Experimental Research

To comprehensively evaluate the tracking accuracy and stability of the proposed tube-MPC method, LQR control and MPC control were selected as benchmark references for comparison.

#### 4.1.1. Trajectory Tracking Accuracy Comparison

**Figure 5:**
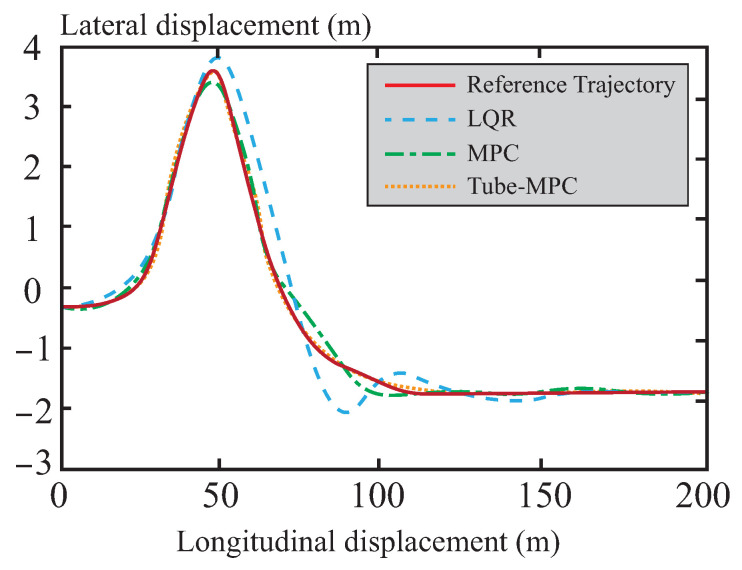
Comparison of global tracking accuracy.

**Figure 6:**
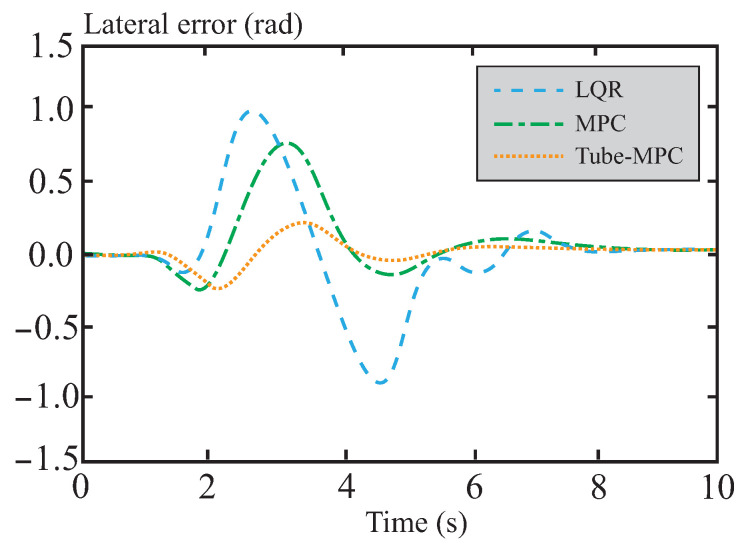
Lateral error comparison.

A comparison and analysis of the control effectiveness of the three methods were conducted using a double lane change scenario. As can be seen from Figure 5, the simulation verifies the effectiveness of all three control methods under this condition, with each capable of tracking the reference trajectory. In Figure 6, it can be observed that the lateral stability of the vehicle is significantly improved under the control of the tube-MPC algorithm. This enhancement is primarily attributed to the introduction of the multi-power reaching law DSMC method. By adaptively adjusting the multi-power term coefficients, this method demonstrates stronger robustness in suppressing system disturbances, thereby achieving higher precision in trajectory tracking. Furthermore, the design of the multi-power sliding mode reaching law further optimizes the dynamic performance of the system, accelerating the convergence speed of states and enabling the system to reach a steady state in a shorter time. This not only enhances the responsiveness of the control system but also improves its real-time performance.

#### 4.1.2. Comparison of Vehicle Stability

**Figure 7:**
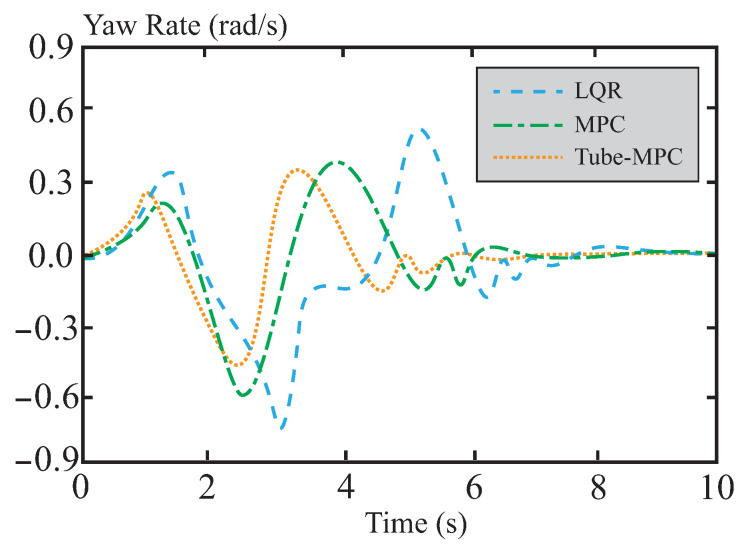
Yaw rate comparison.

**Figure 8:**
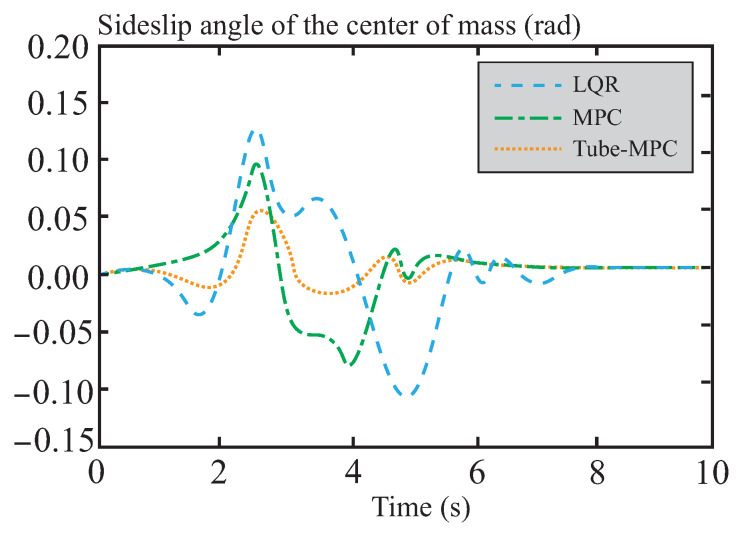
Comparison of centroid lateral declination angle.

Figure 7 and Figure 8 present a performance comparison of different control methods in terms of lateral stability, including two indicators: yaw rate and sideslip angle. In the yaw rate comparison shown in Figure 7, the maximum and minimum values for LQR control are 0.58 rad/s and −0.79 rad/s, respectively, indicating relatively large amplitude oscillations. This reflects its weaker ability to suppress disturbances and its suboptimal dynamic response. For MPC control, the maximum and minimum values are approximately 0.44 rad/s and −0.64 rad/s, respectively, showing a reduction in amplitude. This suggests that MPC outperforms LQR in terms of disturbance rejection, although its oscillation amplitude remains relatively large. The performance of tube-MPC control is even more outstanding, with maximum and minimum values of approximately 0.40 rad/s and −0.47 rad/s, respectively. Its amplitude is significantly reduced, and its response curve converges rapidly, verifying its stronger disturbance rejection capability and dynamic performance. Figure 8 shows that tube-MPC also achieves smoother sideslip angle control. This improvement is due to the robust DSMC framework, which reduces disturbance-induced fluctuations, and an optimized multi-power reaching law that effectively suppresses chattering. As a result, tube-MPC can rapidly adapt its strategy for precise vehicle control under dynamic driving conditions.

**Figure 9:**
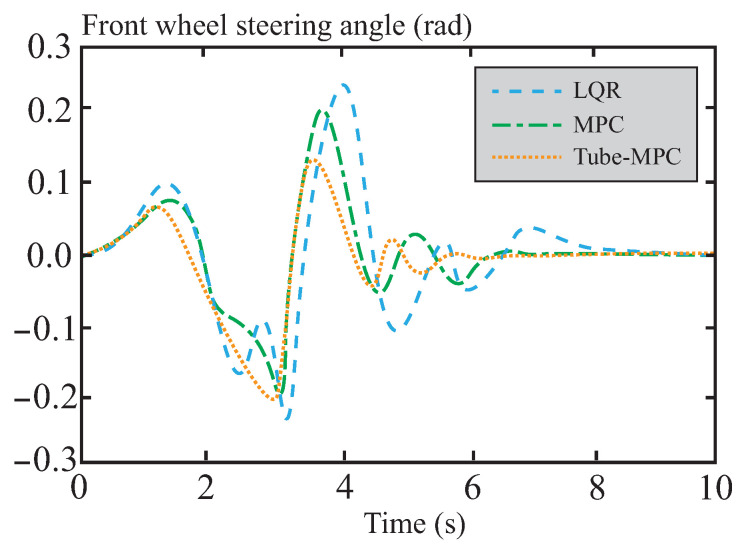
Comparison of front wheel angle.

Figure 9 shows the dynamic response comparison of the front wheel steering angle under the three control methods. The response curve of the LQR control method exhibits significant fluctuations, and its control strategy struggles to effectively suppress disturbances under complex dynamic conditions. In contrast, the response curve of tube-MPC shows the smallest fluctuations and the fastest convergence speed. This improvement is attributed to the CAFNN model, which incorporates historical state and control data in the input layer. By comprehensively considering vehicle state data, tube-MPC gains a better understanding of the dynamic characteristics under different road conditions, thereby significantly enhancing the accuracy and adaptability of dynamic adjustments.

**Figure 10:**
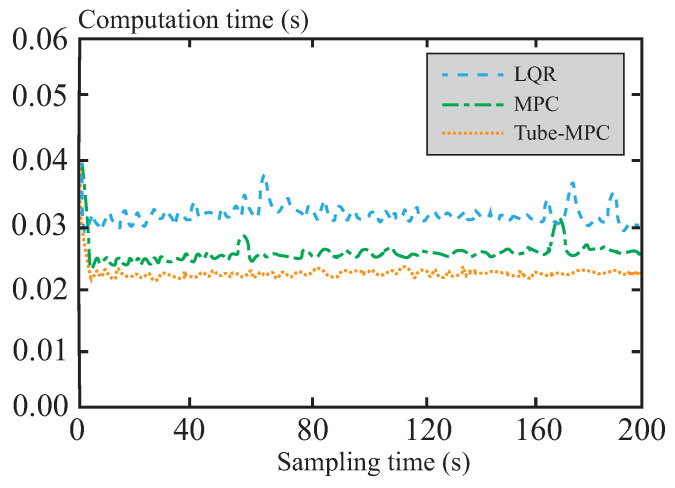
Comparison of computation time.

Figure 10 compares the computation times of the three control methods, clearly demonstrating the performance advantage of tube-MPC. The LQR control method exhibits an overall longer computation time, which may lead to insufficient control precision and robustness in complex working conditions. The traditional MPC method requires directly solving the QP problem, resulting in relatively high computation time, particularly during the first iteration. This is due to the large control horizon and the complex constraint dimensions, causing a significant peak in computation time. In contrast, tube-MPC utilizes the analytical properties of LCP to transform the QP problem into a piecewise function form. By reducing the complexity of the solution, it significantly decreases computation time and resource consumption. This optimized framework ensures real-time performance and efficiency while maintaining good support for control accuracy and system robustness. The experimental results show that tube-MPC has the shortest computation time and the best stability, demonstrating superior overall performance under complex and variable working conditions.

**Figure 11:**
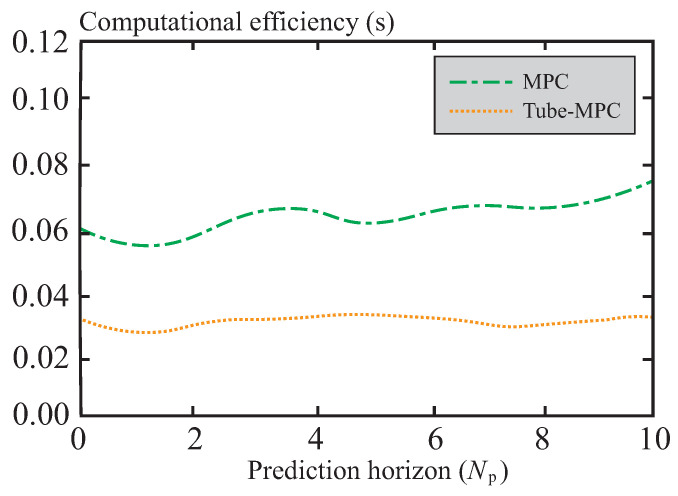
Comparison of computational efficiency.

Figure 11 presents a comparison of computational efficiency between the MPC and tube-MPC methods under different prediction horizons. It can be observed that as the prediction horizon increases, the computational efficiency of MPC exhibits fluctuations, while the computational efficiency of tube-MPC remains relatively stable at around 0.03 s, demonstrating favorable stability. Therefore, the tube-MPC method proposed in this chapter not only achieves stable computational efficiency but also enables rapid convergence of trajectory errors within a short time, showcasing excellent real-time performance.

In the aforementioned research, we have verified the excellent performance and robustness of the tube-MPC algorithm proposed in this paper in trajectory tracking control simulations. However, the simulation environment is ultimately different from actual operating conditions. Therefore, the next step is to deploy this algorithm on a real vehicle platform for experimental validation.

### 4.2. Real Vehicle Experimental Research

To validate the actual performance of the designed algorithm, we conducted real-vehicle experiments on an open road near the test site. The experimental route includes a typical obstacle avoidance section, which can simulate the lane-changing maneuvers that an autonomous vehicle might perform in emergency situations, holding significant research value. Furthermore, comparative experiments were carried out under three different road surface conditions: normal road, muddy road, and snowy road, to comprehensively evaluate the adaptability and robustness of the algorithm in complex and variable real-world driving environments.

#### 4.2.1. Normal Road

**Figure 12:**
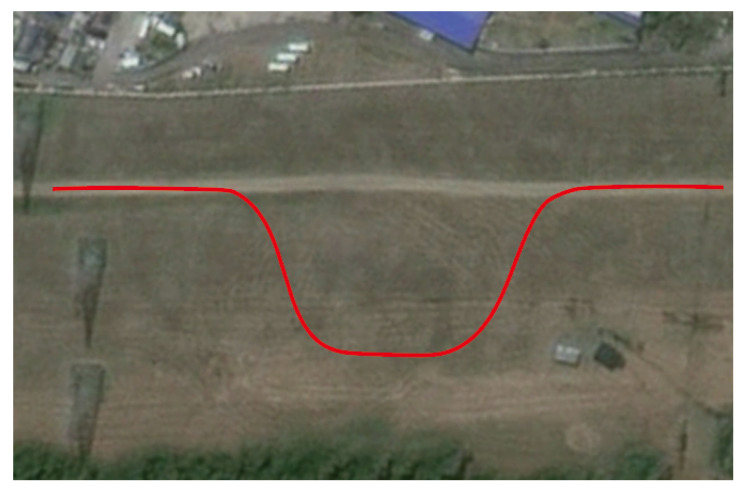
Reference trajectory in normal road.

Figure 12 shows the actual scene of the normal road test section. The section features a smooth surface with good adhesion coefficient, providing a baseline scenario for algorithm testing under standard conditions. Its path characteristics—combining straight segments and gentle curves—align with the typical operating conditions encountered by autonomous vehicles in daily operation, offering favorable experimental conditions for algorithm validation.

**Figure 13:**
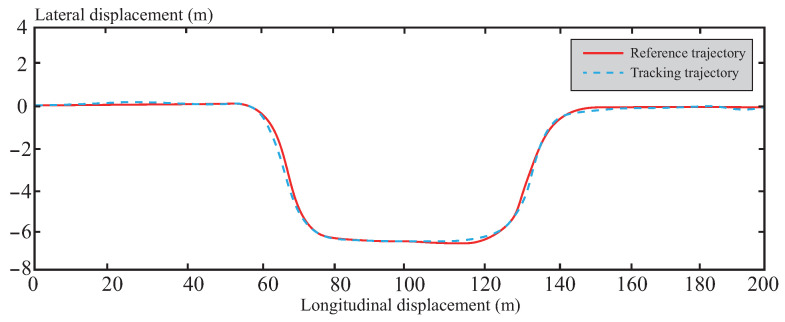
Trajectory tracking effect under normal road conditions.

**Figure 14:**
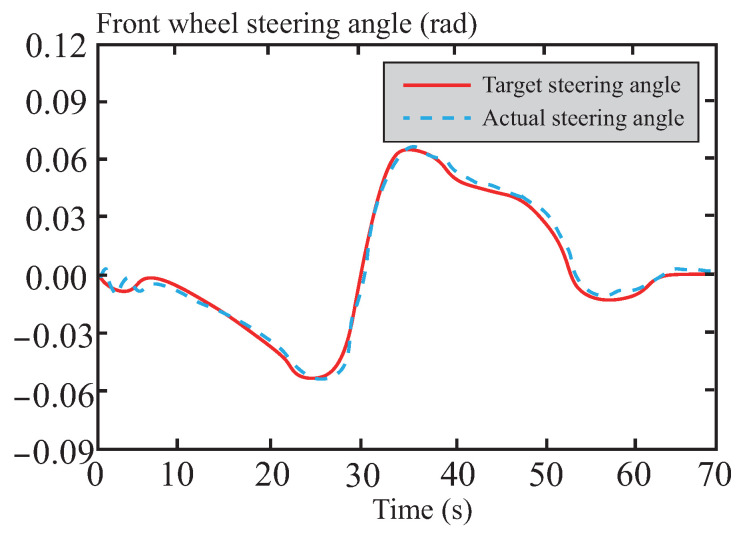
Front wheel angle following curve in normal road.

**Figure 15:**
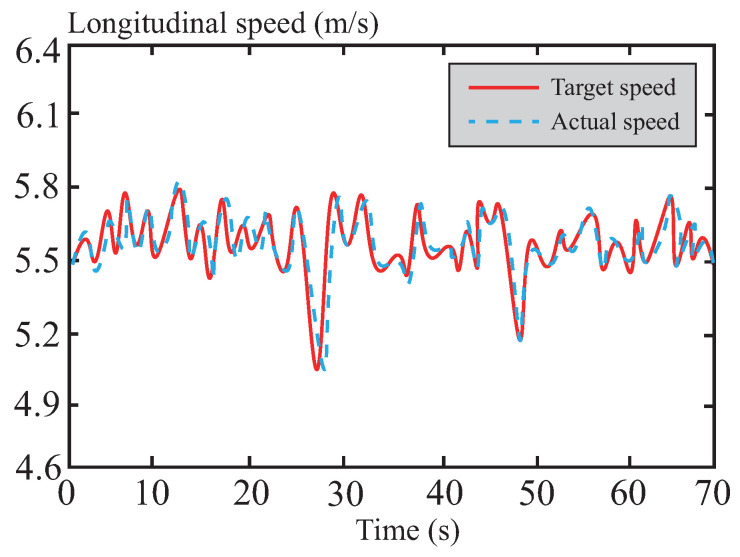
Longitudinal velocity following curve in normal road.

**Figure 16:**
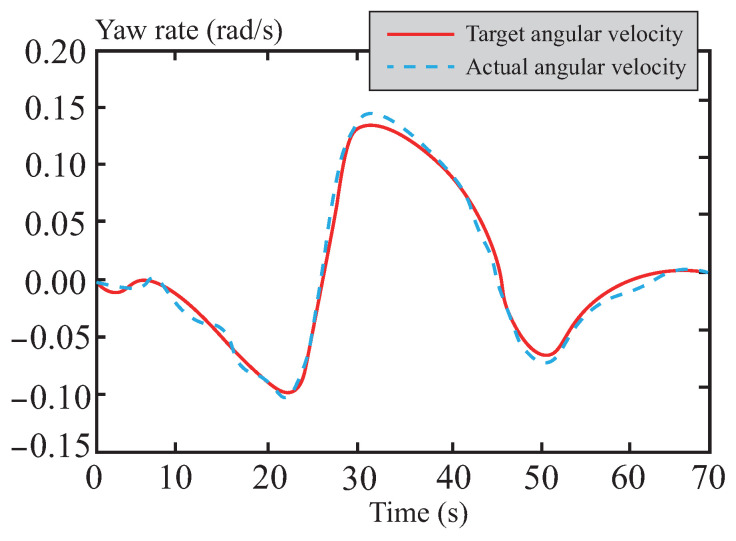
Yaw rate following curve in normal road.

**Figure 17:**
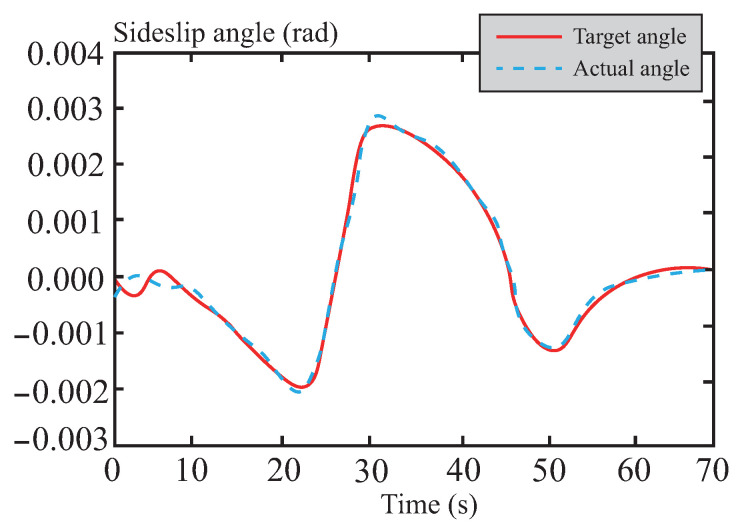
Center of mass lateral declination angle following curve in normal road.

Figure 13, Figure 14, Figure 15, Figure 16 and Figure 17 present the trajectory tracking results of the experiment. The experimental validation demonstrates that the tracking algorithm designed in this paper can achieve high-precision tracking of the desired path under various road conditions, with tracking errors consistently maintained within an acceptable range. As shown in Figure 13, on straight road sections, the vehicle’s tracking trajectory essentially overlaps with the reference trajectory, exhibiting high tracking accuracy. This is attributed to the CAFNN model’s precise identification of the vehicle’s dynamic characteristics, effectively capturing the dynamic behavior during straight-line driving. In curved road sections, due to the significant increase in path curvature and the complexity of vehicle dynamics, some deviation occurs between the tracking trajectory and the reference trajectory. However, the robust optimization framework of tube-MPC effectively suppresses the accumulation of deviations when facing external disturbances, mitigating the control errors caused by increased curvature to some extent and keeping the deviations within an acceptable range. A detailed comparison of the path tracking performance is provided in Table 6.

Based on the results shown in Figure 14, it can be observed that the front wheel steering angle control input remains moderate during the path tracking process, effectively avoiding frequent or drastic steering adjustments. This demonstrates excellent smoothness and strong system robustness. Such outstanding control performance is primarily attributed to the precise optimization solution of the tube-MPC algorithm, which enables rapid and efficient dynamic responses. Furthermore, by leveraging its robust control characteristics, the algorithm successfully suppresses interference from external disturbances and system uncertainties on steering control.

Figure 15 illustrates the variation in the vehicle’s longitudinal speed during the path-tracking process. The curve indicates that the longitudinal speed remains consistently around 20 km/h, showing a high degree of alignment with the preset target speed. This highlights the advantages of the DSMC control within the tube-MPC algorithm, particularly the improved multi-power reaching law strategy adopted in this paper. By significantly enhancing the convergence speed, this strategy accelerates the dynamic response of the control system, effectively suppressing the chattering phenomenon typically associated with traditional DSMC while maintaining excellent control accuracy. Furthermore, this approach further reduces amplitude fluctuations, ensuring the smoothness and robustness of the vehicle’s longitudinal speed. This enables the system to quickly adapt to dynamic changes under complex driving conditions, avoiding speed fluctuations or overshoot phenomena, thereby improving both the accuracy of trajectory tracking and driving stability.

Figure 16 and Figure 17 depict the tracking curves of the vehicle’s yaw rate and sideslip angle. Thanks to the optimization strategy of the tube-MPC algorithm, the control input of the front-wheel steering angle exhibits exceptional smoothness. This characteristic, in turn, significantly and positively influences the dynamic responses of both the yaw rate and the sideslip angle, achieving an optimized control effect. The analysis results indicate that the applied tube-MPC algorithm can effectively suppress potential instabilities during trajectory tracking while substantially enhancing the overall dynamic stability and tracking accuracy of the system. Consequently, it ensures the precision of the control process.

#### 4.2.2. Muddy Road

**Figure 18:**
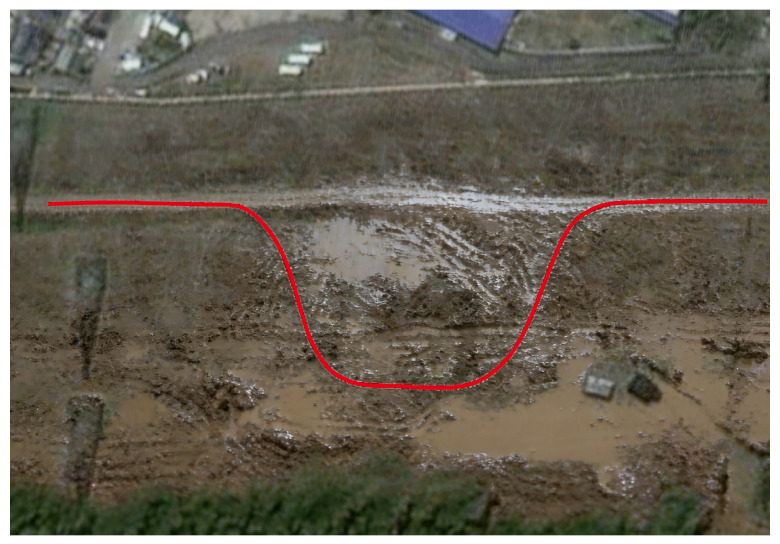
Reference trajectory in muddy road.

Figure 18 shows the real scene of the muddy road test section. With its slippery surface, significantly reduced adhesion conditions, and localized water accumulation, this section highly replicates the actual operating conditions of low adhesion and complex tire-road interaction that autonomous vehicles may encounter. Such scenarios pose severe challenges to vehicle control execution and are particularly effective for testing the adaptability and stability of algorithms under extreme conditions.

**Figure 19:**
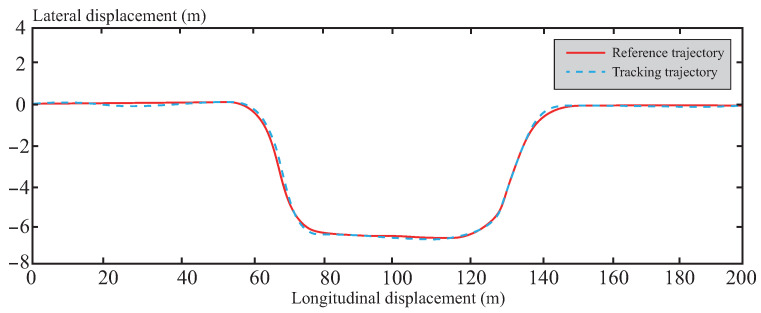
Trajectory tracking effect under muddy road conditions.

**Figure 20:**
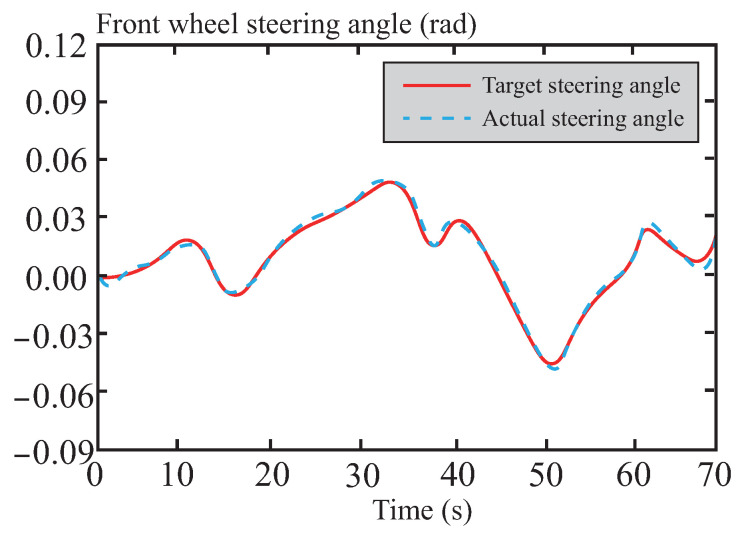
Front wheel angle following curve in muddy road.

**Figure 21:**
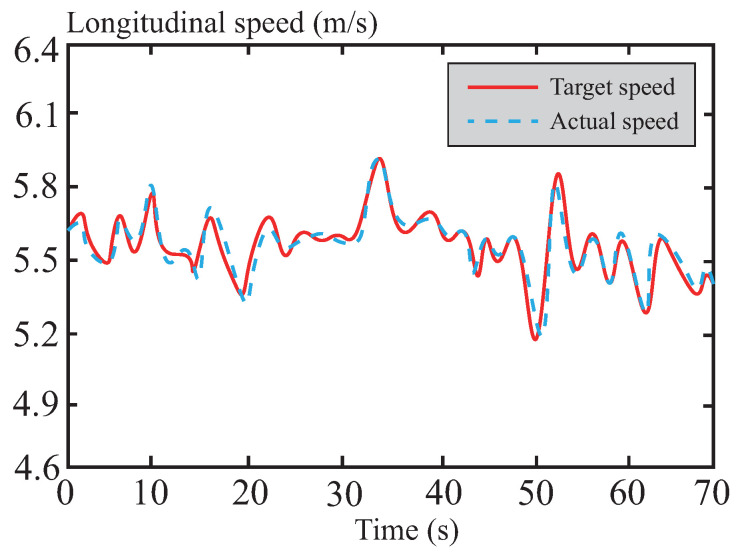
Longitudinal velocity following curve.

**Figure 22:**
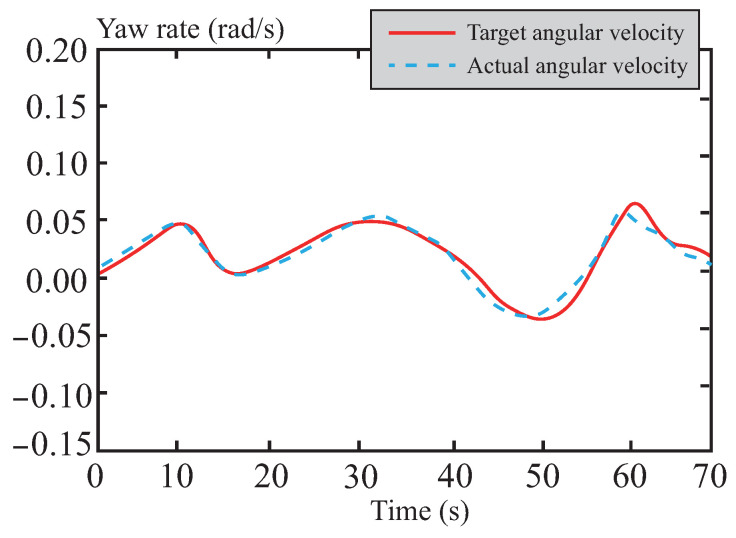
Yaw rate following curve in muddy road.

**Figure 23:**
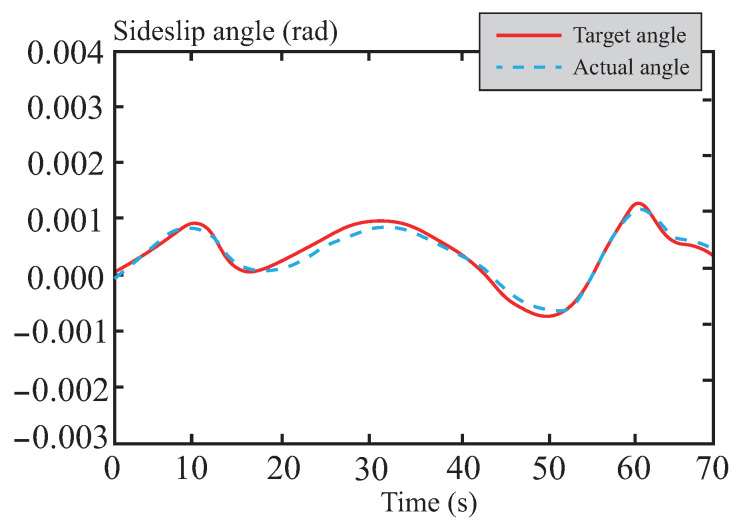
Center of mass lateral declination angle following curve in muddy road.

Figure 19, Figure 20, Figure 21, Figure 22 and Figure 23 present the trajectory tracking results under muddy road conditions. The experiments demonstrate that the tracking algorithm effectively follows the desired path despite low adhesion and complex tire-road interactions. As shown in Figure 19, on straight muddy sections, the vehicle’s trajectory closely matches the reference despite reduced surface adhesion, highlighting the algorithm’s straight-line stability. This is achieved through the CAFNN model, which accurately identifies nonlinear vehicle dynamics on low-adhesion surfaces, improving predictions for longitudinal and lateral control. In muddy curves, where adhesion decreases further and water patches create uneven conditions, path curvature and vehicle dynamics become highly complex. The tube-MPC framework, with its predefined disturbance sets and state constraints, absorbs uncertainties like sudden surface changes and tire saturation, effectively limiting deviations and maintaining vehicle controllability under extreme low-adhesion conditions. Table 7 provides a detailed analysis of the path-tracking effect under muddy road conditions.

According to the results shown in Figure 20, it can be observed that during path tracking on the muddy road section, the front wheel steering angle control input remains relatively stable despite being affected by low adhesion and road surface disturbances. This effectively suppresses unstable steering adjustments caused by road surface slip and uncertainties, demonstrating the control robustness of the algorithm under harsh conditions. Moreover, the algorithm mitigates the impact of complex factors, such as sudden changes in road adhesion and wheel slip, on steering control.

Figure 21 shows the variation of the vehicle’s longitudinal speed during path tracking on muddy roads. From the curve, it can be observed that despite the slippery surface and uneven adhesion conditions caused by water accumulation, the vehicle’s longitudinal speed remains stable around the target value of 20 km/h. Minor instantaneous fluctuations occur only in local sections due to tire slip or sudden changes in road surface conditions, while overall consistency with the preset target speed is maintained. Compared to normal road conditions, the dynamic characteristics of muddy road sections are more complex. The proposed strategy in this study optimizes the reaching law structure, effectively reducing the amplitude of chattering in control outputs while maintaining strong system robustness and significantly improving the system’s dynamic convergence speed under abrupt changes in adhesion. Particularly during brief wheel slip, the controller can quickly adjust the driving and braking torque, suppressing sustained fluctuations in longitudinal speed and maintaining the overall stability of the vehicle’s motion.

Figure 22 and Figure 23 present the tracking curves of the yaw rate and sideslip angle during vehicle operation on muddy roads. Influenced by low adhesion and uneven surface disturbances, the vehicle’s lateral dynamics become more complex, imposing stricter demands on steering control. The algorithm provides stable control without significant chattering or instability caused by sudden adhesion changes or slipping. Experimental results show that the control framework reduces coupled oscillations between sideslip and yaw motion through real-time optimization and disturbance compensation. This maintains path-tracking accuracy while improving overall handling safety and controllability on low-adhesion surfaces.

#### 4.2.3. Snowy Road

**Figure 24:**
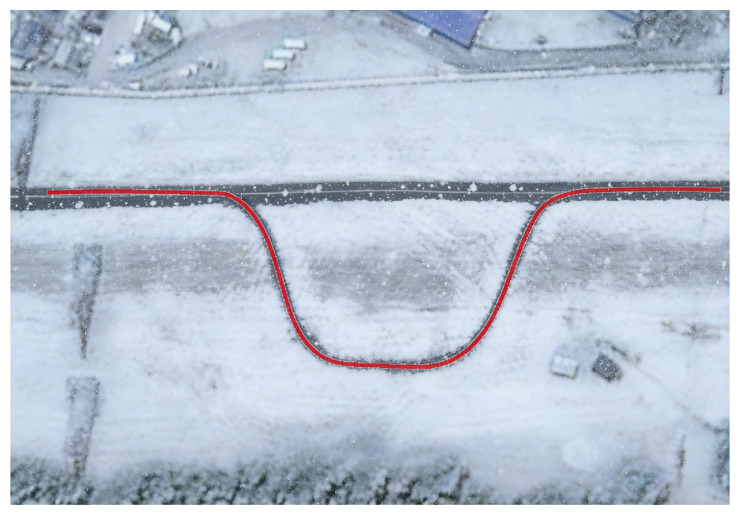
Reference trajectory in snowy road.

Figure 24 shows the actual scene of the snowy road test section. With its snow-covered surface, significantly reduced adhesion coefficient, and presence of icy patches and uneven compacted snow layers, this section highly simulates the real-world driving conditions that autonomous vehicles may encounter in cold climates—characterized by extremely low adhesion and the impact of low temperatures on vehicle performance.

**Figure 25:**
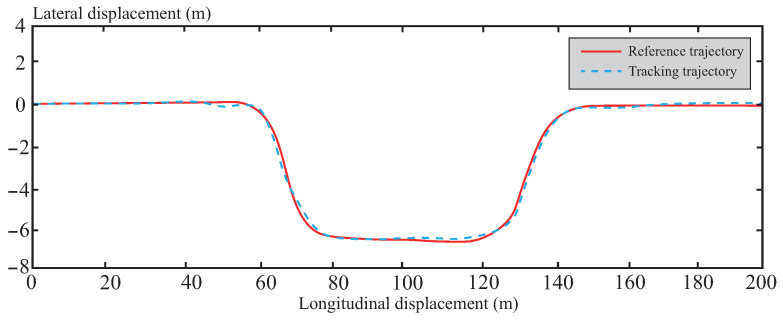
Trajectory tracking effect under snowy road conditions.

**Figure 26:**
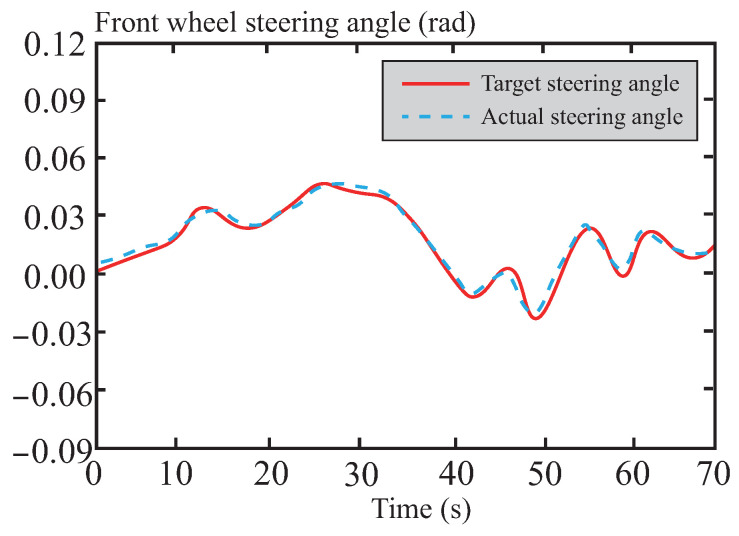
Front wheel angle following curve in snowy road.

**Figure 27:**
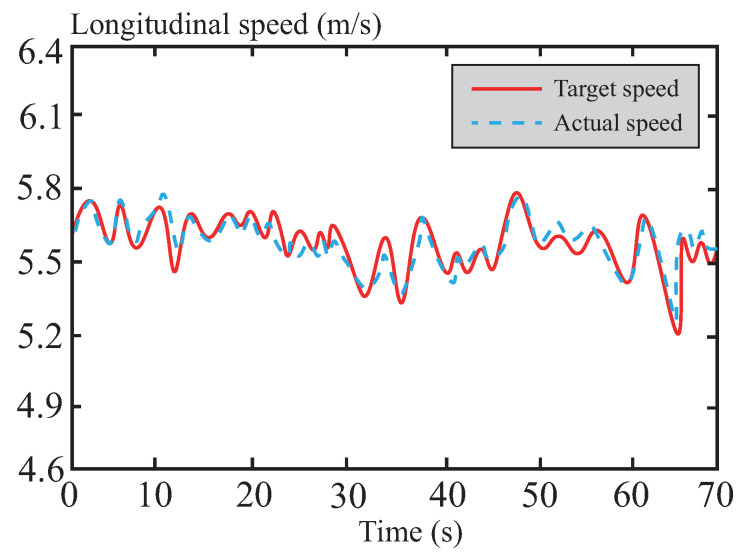
Longitudinal velocity following curve in snowy road.

**Figure 28:**
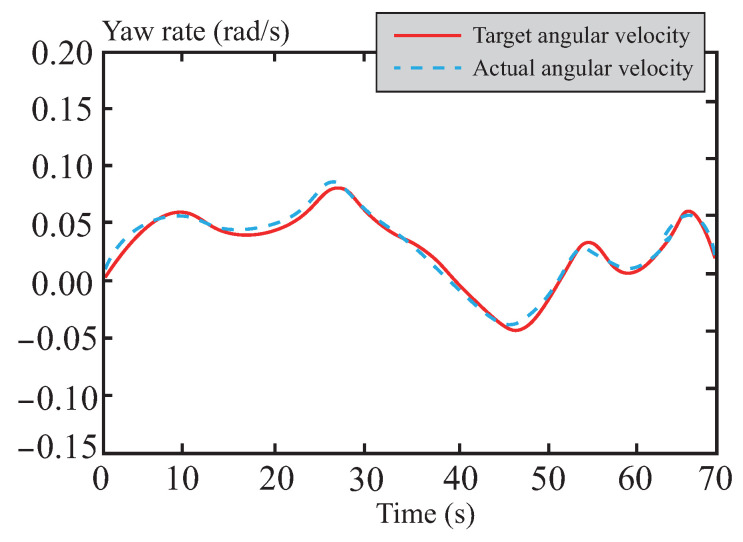
Yaw rate following curve in snowy road.

**Figure 29:**
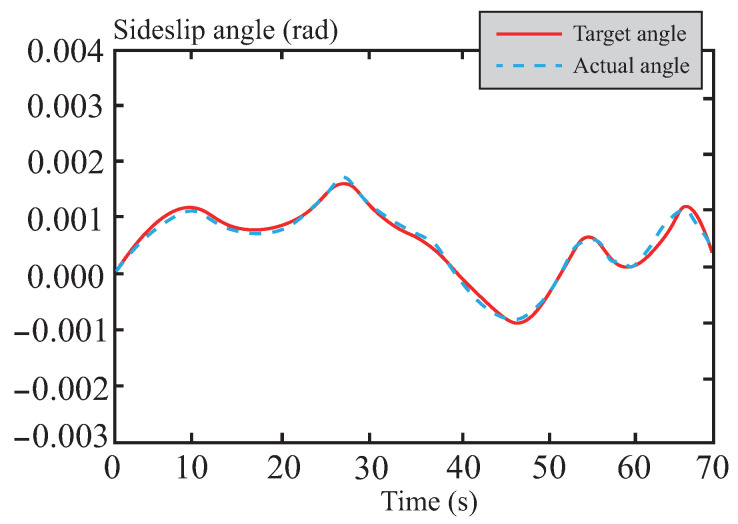
Center of mass lateral declination angle following curve in snowy road.

Figure 25, Figure 26, Figure 27, Figure 28 and Figure 29 present the experimental results of trajectory tracking on snowy roads. The experiments demonstrate that the tracking algorithm designed in this paper can achieve stable tracking of the desired path under icy and snowy conditions characterized by low adhesion coefficients and uneven road surface properties. As shown in Figure 25, on straight snowy road sections, even under the dual influence of significantly reduced road adhesion and localized icing, the vehicle’s tracking trajectory remains largely consistent with the reference trajectory, demonstrating excellent straight-line tracking stability. On curved snowy road sections, snow and ice cause a sharp decrease in road adhesion, resulting in controllable and reasonable deviations between the actual tracking trajectory and the reference path. In response, the robust optimization framework of tube-MPC effectively counteracts error accumulation caused by abrupt changes in adhesion, tire slip, and actuator response delays in low-temperature environments. This significantly mitigates the control deviations caused by the coupling effect of road curvature and low-adhesion surfaces, ensuring stability and controllability during vehicle steering maneuvers. Table 8 provides a detailed analysis of the path-tracking performance on snowy roads.

According to the results shown in Figure 26, during snowy road path tracking, the control input of the front wheel steering angle remains relatively stable despite facing challenges such as drastic changes in road adhesion and nonlinear coupling. It does not produce severe fluctuations due to sudden adhesion changes or local slipping, demonstrating good smoothness and environmental adaptability. This stability performance benefits from the algorithm’s robust optimization under high uncertainty, which achieves fast and accurate control responses in complex low-temperature and low-adhesion conditions through real-time rolling optimization and constraint handling. At the same time, its built-in disturbance suppression mechanism effectively mitigates composite interferences such as road unevenness and transient changes in traction, enhancing the reliability of trajectory tracking under extreme operating conditions.

Figure 27 shows the longitudinal speed variation of the vehicle during path tracking on snowy roads. The experimental results indicate that, even on low-adhesion icy and snowy surfaces, the vehicle’s longitudinal speed remains stably maintained around the target value of 20 km/h, demonstrating excellent speed tracking performance. This highlights the effectiveness of the proposed algorithm in addressing challenges posed by low-temperature environments, particularly in overcoming the impact of mechanical characteristic changes in the vehicle due to low temperatures on the control system. Consequently, the algorithm significantly enhances the control smoothness of the system under extreme operating conditions.

Figure 28 and Figure 29 present the tracking curves of the vehicle’s yaw rate and sideslip angle on snowy roads, respectively. Addressing the critical challenge of vehicle instability caused by low-adhesion surfaces in snowy conditions, the optimized front-wheel steering angle control input from the tube-MPC algorithm demonstrates excellent smoothness. This characteristic significantly improves the dynamic responses of both yaw rate and sideslip angle, achieving high-precision control performance on snowy roads. The results indicate that the algorithm effectively suppresses slip-induced instability risks during trajectory tracking, substantially enhances the system’s dynamic stability and tracking accuracy, and ensures both precision and robustness in the control process under low-adhesion road conditions.

The proposed algorithm exhibits robust control performance in critical states such as front-wheel steering limit and tire adhesion saturation during real-vehicle tests: the auxiliary SMC controller in the tube-MPC framework will adjust the control output in real time to avoid steering actuator saturation, and the CAFNN model pre-identifies the tire nonlinear characteristics under low friction, effectively suppressing the control deviation caused by tire saturation. The tested road conditions cover the friction coefficient range of 0.2 (snowy road)∼0.85 (normal road), and the algorithm maintains stable tracking at the critical speed of 25 km/h for low-adhesion roads, without trajectory divergence or control failure.

In summary, the tube-MPC control algorithm based on CAFNN identification demonstrates excellent trajectory tracking performance in obstacle avoidance scenarios under three different road surface conditions. On straight road sections, the system accurately follows the reference trajectory, ensuring high precision and stability in path tracking. On curved road sections, due to high curvature and complex vehicle dynamics, some deviations occur between the reference trajectory and the actual trajectory. However, these deviations consistently remain within acceptable limits. The yaw rate and front-wheel steering control inputs exhibit smooth responses without any instability, further validating the algorithm’s reliability in terms of dynamic stability. By integrating the precise modeling capabilities of CAFNN with the robust control characteristics of the tube-MPC algorithm, the system not only responds swiftly to external disturbances but also significantly enhances dynamic adaptability and control accuracy during path tracking. Moreover, the optimization method combining QP-MPC with LCP effectively reduces computational complexity, improving real-time control efficiency. Additionally, the improved DSMC strategy, incorporating a multi-power reaching law, accelerates convergence speed while suppressing the chattering phenomenon common in conventional DSMC, ensuring stable and reliable performance even during high-curvature trajectory tracking. The experimental results fully validate the algorithm’s comprehensive performance in handling complex vehicle dynamics, high-curvature path tracking, and varying operating conditions, while demonstrating its feasibility and robustness in practical application environments.

## 5. Conclusions

This paper investigates the modeling and control of vehicle lateral kinematics based on an improved fast tube-MPC method. A neural network kinematic model is designed based on CAFNN, leveraging the control structure of affine systems and the powerful learning capability of neural networks to effectively address nonlinearities and uncertainties, thereby improving global accuracy. For the control problem, a fast tube-MPC controller is designed, where the MPC controls the nominal system and the SMC controls the error system. To enhance the system’s rapidity, the MPC problem is linked to the LCP via the ramp function, enabling the rapid computation of the QP problem solution using PWA functions. To improve the system’s robustness, a multi-power sliding mode reaching law is designed, which introduces adjustable power term coefficients compared to the single-power sliding mode reaching law, accelerating the system’s convergence speed to better handle uncertainties and external disturbances within the system. Finally, simulations under different operating conditions and road adhesion coefficients validate the effectiveness of the proposed method. In the future, we will further explore the deployment and optimization of this framework on real-time embedded platforms, investigate its integration strategies with vehicle state estimation and environmental perception modules to build a more comprehensive intelligent driving control system.

## Figures and Tables

**Table 1:** Quantitative indicators of CAFNN model identification accuracy.

Evaluation Metric	Training Set	Test Set
$x_{h}$ (RMSE)	0.021	0.035
$y_{h}$ (RMSE)	0.018	0.029
θ (RMSE)	0.003	0.005
$v_{h}$ (RMSE)	0.042	0.061
ω (RMSE)	0.012	0.018
β (RMSE)	0.002	0.004
Comprehensive (MAE)	0.019	0.028
Comprehensive ($R^{2}$)	0.987	0.972

**Table 2:** Performance comparison between CAFNN and benchmark models.

Model	Comprehensive RMSE	Training Time	Real-Time Inference Time
Traditional model	0.127	none	0.001 ms
BP neural network	0.053	12.8 s	0.008 ms
CAFNN model	0.031	15.3 s	0.009 ms

**Table 3:** Comparison of computational performance between two different solution methods.

Solution Method	Average Computation Time (ms)	Maximum Computation Time (ms)
Proposed method	2.36	2.89
Traditional QP	8.72	12.53

**Table 4:** Controller parameters of the fast tube-MPC algorithm.

Nominal Controller	Parameters	Auxiliary Controller	Parameters
$N_{p}$	10	*C*	{1,1,5,5,5,5}
$N_{c}$	5	κ	{0.15,0.25,0.25,0.15,0.15,0.1}
*Q*	diag{1,1,1,1,1,1}	$k_{1}$	0.8
*R*	5	$k_{2}$	1
*P*	1000	$k_{3}$	1.2
$v_{max}$	40	α	1.9
$\delta_{max}$	30	β	0.5

**Table 5:** Basic parameters of real vehicles.

Vehicle Parameters	Value
Total vehicle mass m/[kg]	1300
Vehicle moment of inertia about the z-axis $I_{z}$/[kg·m^2^]	2286
Distance from the front axle to the vehicle center of mass *a*/[m]	1.51
Distance from the rear axle to the vehicle center of mass *b*/[m]	1.59
Front wheel cornering stiffness $C_{f}$/[N/rad]	57,810
Rear wheel cornering stiffness $C_{r}$/[N/rad]	50,392
Track width [m]	12.9
Maximum speed [km/h]	120
Range [km]	150

**Table 6:** Comparison of trajectory tracking performance under normal road conditions.

Category	Average Deviation	Maximum Value	Minimum Value	Standard Deviation
Straight section	0.178	0.129	−0.285	0.137
Curved section	0.235	0.273	−0.326	0.184

**Table 7:** Comparison of path following effect under muddy road conditions.

Category	Average Deviation	Maximum Value	Minimum Value	Standard Deviation
Straight section	0.189	0.145	−0.306	0.144
Curved section	0.273	0.265	−0.356	0.189

**Table 8:** Comparison of path following effect under snowy road conditions.

Category	Average Deviation	Maximum Value	Minimum Value	Standard Deviation
Straight section	0.207	0.213	−0.407	0.225
Curved section	0.304	0.289	−0.301	0.175

## Data Availability

The data used to support the findings of this study are available from the corresponding author upon request.
